# Supplementary material for: Oriented intergrowth of the catalyst layer in membrane electrode assembly for alkaline water electrolysis
Source: Nat Commun. 2022 Dec 27;13:7956. doi: 10.1038/s41467-022-35603-9 (PMC9794718; doi:10.1038/s41467-022-35603-9)
Supplement: Supplementary file 1 — Supplementary Information [file 41467_2022_35603_MOESM1_ESM.pdf]

Supplementary Information

Oriented intergrowth of the catalyst layer in membrane electrode assembly for alkaline water electrolysis

Lei Wan[1], Maobin Pang[1], Junfa Le[1], Ziang Xu[1], Hangyu Zhou[1], Qin Xu[1] and Baoguo Wang*[1]

[1]Department of Chemical Engineering, Tsinghua University, Beijing, China

*Corresponding author. Email: bgwang@tsinghua.edu.cn

Supplementary Notes

1. Experimental & Results

1.1. Basic property of membranes characterization

Water contact angle measurement. Samples of all-in-one MEAs and other membranes were cut into ~2 cm × 2 cm pieces and immersed in deionized water to reach a fully wet state before water contact angle measurement (Dataphysics OCAH200). A setting of 10 μL volume drop deionized water was used at room temperature.

Electrolyte uptake and swelling measurement. To measure the electrolyte uptake (including water and KOH) and swelling ratio of the all-in-one MEAs, the weight and length of membranes were measured before treating with 30 wt.% KOH and after immersing in 30 wt.% KOH, respectively. To measure the weight of dry membranes, the membranes were dried in a vacuum at 60 ℃ for 24 h. The electrolyte uptake of the different membranes was defined as: electrolyte uptake = $(m_h - m_d)/m_d \times 100\%$, in which m_d and m_h are the mass of dty and hydrated membranes, respectively. The swelling ratio of the different membranes was defined as: swelling ratio = $(X_w - X_d)/X_d \times 100\%$, in which X_d and X_w are the length of corresponding dry and wet membranes, respectively.

Area resistance measurement. The area resistance of the membranes was evaluated by a two-electrode electrochemical impedance spectroscopy (EIS) method in an assembled coin cell. The potentiostat with an AC bias and a frequency range were set 10 mV and 1 MHz-0.1 Hz, respectively. The samples impregnated with aqueous electrolyte (10-30 wt.% KOH) were sandwiched between two electrodes with an avtive area of 1.13 cm^{-2}, and sealed with coin cell cases for area resistance measurement at 25 ℃.

The hydroxide ions transport properties. The ionic transport properties of different membranes were investigated using a electrochemical workstation (VersaSTAT-3F, Princeton Applied Research). The current–voltage (I–V) profile was recorded when the membrane was sandwiched between two cells soaking with a gradient of 1–3 mol L^{-1} NaOH solution. Two Ag/AgCl reference electrodes filled with saturated KCl solution and two salt bridges filled with saturated KCl solution were employed to eliminate the potential

drop. Thus, the open-cell voltage of the device (V_0) is equal to the value of diffusion potential (V_d) resulted from the NaOH concentration gradient, which can be calculated as the following equation: $V_0 = V_d = \frac{RT}{F}(t_{Na+} - t_{OH-})\ln(\Delta)$. R, T, F, t_{Na+}, t_{OH-}, and Δ are the gas constant, temperature, faraday constant, Na^+ transference number, OH^- transference number, and activity gradient (the mean ion activity coefficient was considered since the concentration of NaOH solution is high), respectively.

Bubble point pressure measurement. Samples of all-in-one MEAs of 100% relative humidity were cut into 2 cm × 2 cm pieces and equipped into a bubble pressure analyzer. Pressurized Ar gas was applied to the sample. Bubble pressure value was record when the first bubble appeared.

Mechanical strength measurement. The mechanical properties of membranes in dry state were measured using a microcomputer controlled electronic universal testing machine (Changchun Kexin Co.,China) at room temperature in controlled force mode with a stretch rate of 5 mm min^{-1}.

Alkaline stability measurement. A piece of membranes (1 cm × 6 cm) was cut out and immersed into 30 wt.% KOH solution and treated at 80 °C. The area resistance and mechanical strength were measured to evaluate its alkaline stability.

1.2. Overpotential Analysis

The cell voltage E_{cell} is composed of the sum of the reversible cell potential E_{rev} and the three main overpotential η_i:

$E_{cell} = E_{rev} + \eta_{kin} + \eta_{ohm} + \eta_{mass}$

Where η_{kin} is the kinetic, η_{ohm} is the ohmic, and η_{mass} is the mass transport overpotential.

At a temperature of 60 °C, the saturation pressure of H_2O was 0.47 bar_a. For liquid water, the activity of water, a(H_2O), was one, while the activity of the gaseous species was represented by the ratio of their partial pressure to the standard pressure of 1 bar. The temperature dependent standard reversible potential, E_{rev}, could be obtained from the literature

$E_{rev} = 1.2291 - 0.0008456\ (T\text{-}298.15)$

Where the voltages, first two terms on right hand side of equation, were measured in V, and the temperatures in K. Under current electrolyzer testing conditions, the E_{rev} was calculated to be 1.168 V, with a thermoneutral voltage of 1.42 V.

Ohmic overpotential (η_{ohm})

EIS was used to measure the high frequency resistance (HFR) representing the total electronic cell resistance R_{tot}. The ohmic overpotential, η_{ohm}, is therefore determined as:

$\eta_{ohm} = i \times R_{tot} = i \times HRF$

Kinetic overpotential(η_{kin})

The kinetic overpotential was extracted using a Tafel model, in which the Tafel slope *b* and exchange current density i_0 were the governing kinetic parameters. The Tafel model was fitted to iR-free cell voltages between 4 and 20 mA cm^{-1}. Assuming a non-polarizable HER, the entire kinetic overpotential of the cell was governed by OER with the Tafel slope *b* as 2.303 × *RT/4F* where *R* is the ideal gas constant, T is temperature, and F is Faraday's constant:

$\eta_{kin} = b \times \log(i/i_0)$

Mass transport Overpotential(η_{mass})

Mass transport was defined as a sum of gaseous/liquid transfer in the LGDL/CL and ionic transport in the CLs. In this study, it was calculated by subtracting the reversible cell potential and kinetic and ohmic overpotentials from the measured cell potential.

1.3. Levelized cost of hydrogen (LCOH) analysis

The LCOH was used as an index to quantify the cost of hydrogen production from alkaline water electrolysis. The LCOH ($ kg^{-1}) can be expressed as follows:

$$\mathrm{LCOH} = \frac{(\mathrm{CAPEX} \times \mathrm{CRF}) + C_{O\&M}}{M_{H2}}$$

Where CAPEX is the total capital expenditure of the electrolyzer ($), CRF is the capital recovery factor, $C_{O\&M}$ is the annual operation and maintenance costs ($) and M_{H2} is the total hydrogen produced by the electrolyzer in one year (kg).

CRF stands for the capital recovery factor, which converts the capital cost into a series of equivalent annual payments over the system lifetime N considering an interest rate i.

$$\text{CRF} = \frac{i \times \ (1+i)^N}{(1+i)^N - 1}$$

The capital costs of the electrolyzer CAPEX can be calculated with Eq. (3).

$$\text{CAPEX} = \ P_{el} \times I_{el}$$

Where P_{el} is the rated power of the electrolyzer (kW) and I_{el} is the specific investment cost of the electrolyzer ($ kW^{-1}).

The annual operation and maintenance costs $C_{O\&M}$($) include the costs of electricity, electrolyte, nonfuel variable operation and maintenance.

$$C_{O\&M} = (\tau \times P_{el} \times \mu_{el} \times c_e) + (\gamma \times M_{H2} \times c_w) + (\text{CAPEX} \times \varphi)$$

Where τ is the total number of hours in the year (h), P_{el} is the rated power of the electrolyzer (kW), μ_{el} is the utilization rate of the electrolyzer expressed as a fraction of 1, c_e is the price of electricity ($ kWh^{-1}), γ is the water required to produce each kg of hydrogen (L kg^{-1}), M_{H2} is the hydrogen produced by the installation in one year (kg), and c_w is the price of electrolyte ($ kg^{-1}). Maintenance costs are assumed to be constant throughout the system's lifetime and are estimated as a fraction (φ) of the electrolyzer capital cost.

The annual production of hydrogen using alkaline water electrolysis can be computed using Eq. (5):

$$M_{H2} = \frac{\tau \times P_{el} \times \mu_{el}}{E_{el}}$$

In the equation above, E_{el} stands for the power consumption of the electrolyzer (kWh kg^{-1}).

The energy efficiency of an electrolysis cell is defined as the net energy present in the hydrogen produced by the cell divided by the net energy consumed by the cell to produce it, expressed as a percentage. The net energy present in hydrogen is its higher heating value (HHV), which is 39.4 kWh kg^{-1} of hydrogen. An electrolysis cell operating at a cell potential equal to the the voltage of water electrolysis (1.47 V at 60 ℃) displays 100% energy efficiency. In this work, the energy efficiency of advanced alkaline water electrolysis with conventional MEA-CCM and all-in-one MEA-S is ~84% and ~89% at the current density of 1000 mA cm^{-2}, respectively. Owing to the repid degradation of conventional MEA-CCM, the energy efficiency of alkaline water electrolysis using conventional MEA-CCM is ~70% after the stability test.

Common assumptions:

Advanced alkaline water electrolysis stack system is constructed by stacking 200 single cells (200-cell) (Supplementary Table 5). The stack provided a current density of 1000 mA cm^{-2} at approximately 392, 356, 330 and 314 V_{stack} for conventional MEA-CCM (Pt/C//X37-50//IrO_2), conventional MEA-CCS (CoNiS//Zirfon Thin//CoNiS), all-in-one MEA-S and all-in-one MEA (CoNiS@FeNi LDH), respectively.

Conventional MEA-CCM (Pt/C//X37-50//IrO_2): electricity cost = \$25 MWh^{-1}, the specific investment cost of the electrolyzer I_{el} = \$400 kW^{-1}, electrolyzer lifetime = 10 years, interest rate (i) = 6.5%, operations and maintenance (O&M) cost = 4% of electrolyzer CAPEX p.a., cost of electrolyte (5 wt% KOH) = \$0.002 kg^{-1}.

Conventional MEA-CCS (CoNiS//Zirfon Thin//CoNiS): electricity cost = \$25 MWh^{-1}, the specific investment cost of the electrolyzer I_{el} = \$300 kW^{-1}, electrolyzer lifetime = 20 years,

interest rate (i) = 6.5%, operations and maintenance (O$M) cost = 4% of electrolyzer CAPEX p.a., cost of electrolyte (30 wt% KOH) = $0.01 kg^{-1}.

All-in-one MEA-S and all-in-one MEA (CoNiS@FeNi LDH): electricity cost = $25 MWh^{-1}, the specific investment cost of the electrolyzer I_{el} = $200 kW^{-1}, electrolyzer lifetime = 20 years, interest rate (i) = 6.5%, operations and maintenance (O&M) cost = 4% of electrolyzer CAPEX p.a., cost of electrolyte (30 wt% KOH) = $0.01 kg^{-1}, cost of electrolyte (5 wt% KOH) = $0.002 kg^{-1}.

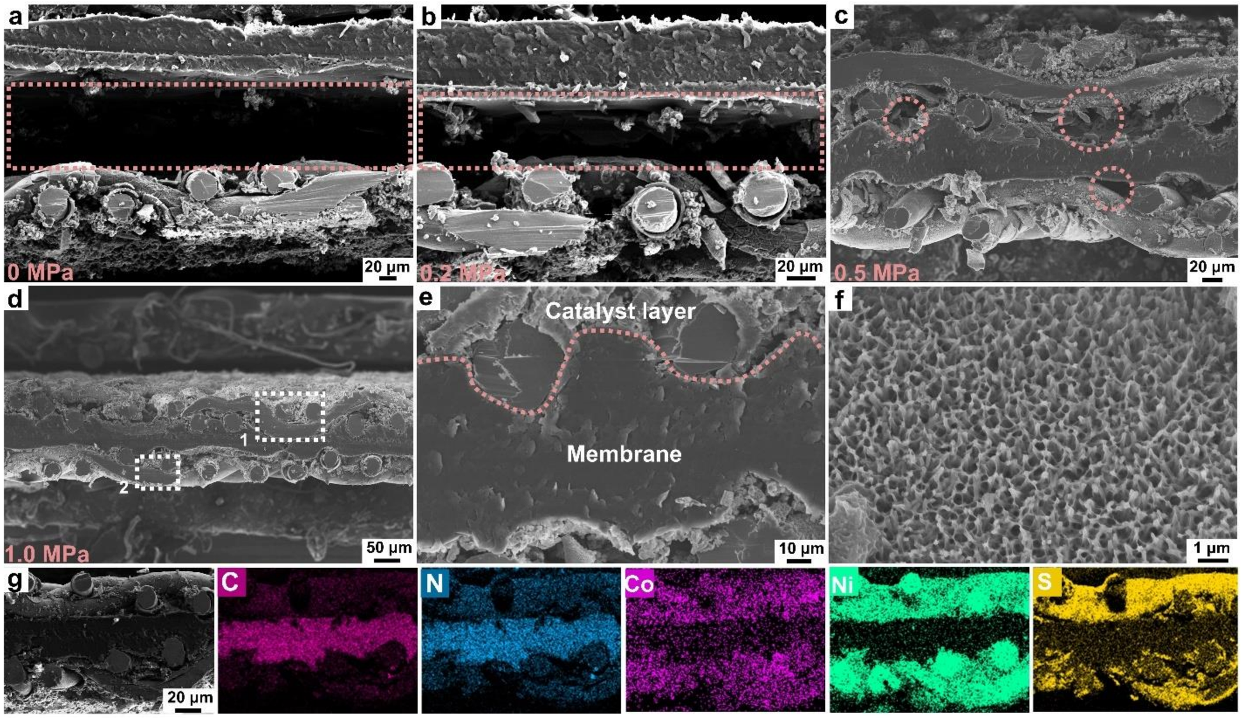

Supplementary Fig. 1 | Morphologies characterization of C-MEA-CCS (CoNiS//X37-50//CoNiS). SEM images of MEAs prepared by hot-pressing of (a) 0 MPa, (b) 0.2 MPa, (c) 0.5 MPa and (d) 1.0 MPa. (e) SEM images of the interface between catalyst layer and membrane (the position 1 marked in (d)). (f) SEM images of catalyst layer (the position 2 marked in (d)). (g) Corresponding EDX mapping of MEA prepared by hot-pressing of 1.0 MPa.

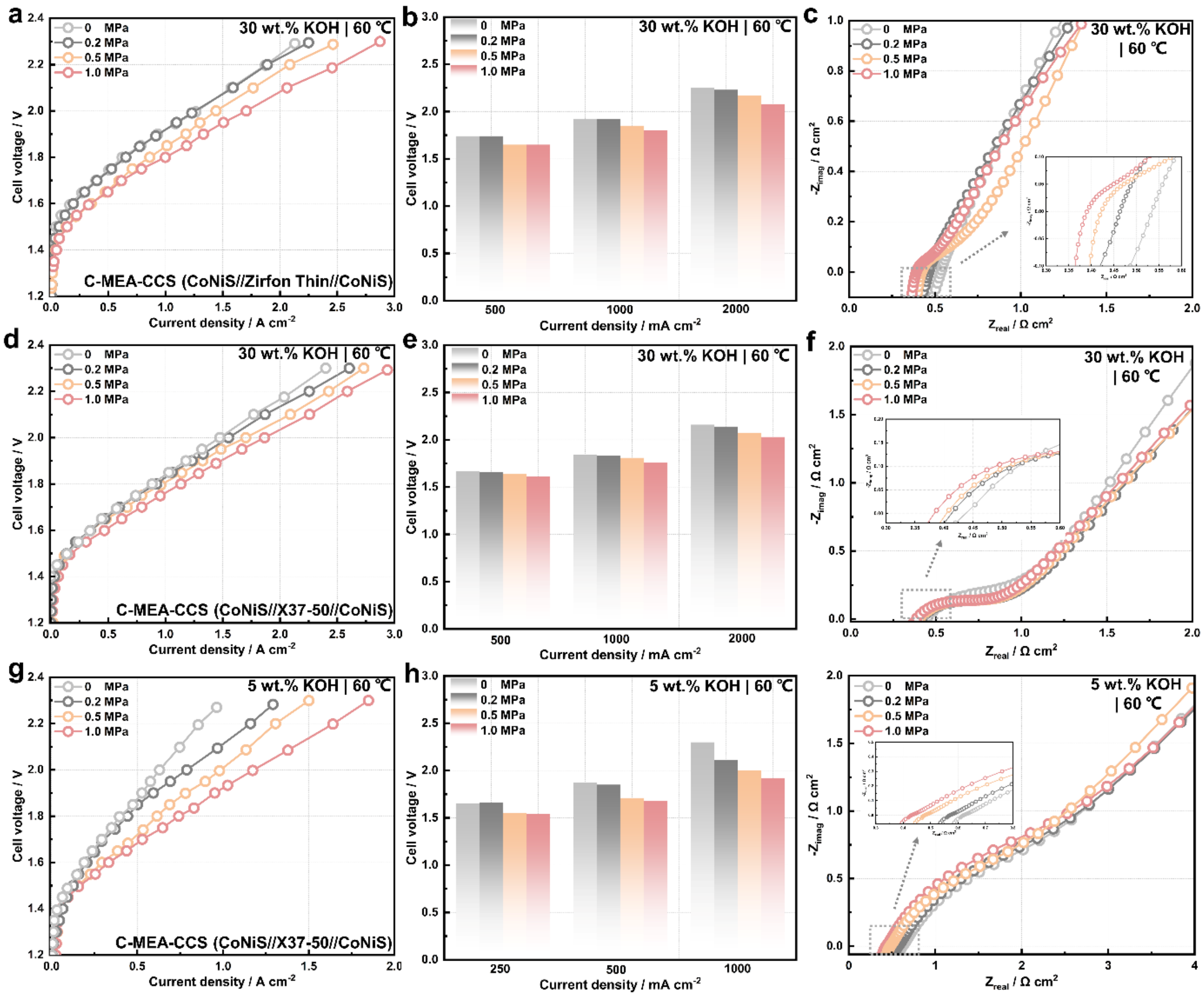

Supplementary Fig. 2 | Performance of MEAs using different hot-pressing pressure. C-MEA-CCS (CoNiS//Zirfon Thin//CoNiS) uses commercial Zirfon Thin (200 μm) and self-supported CoNiS as membrane and electrode, respectively; C-MEA-CCS (CoNiS//X37-50//CoNiS) uses commercail Sustainion X37-50 and self-supported CoNiS as membrane and electrode. There is no ionomer in the cathode and anode. (a) Polarization curves of C-MEA-CCS (CoNiS//Zirfon Thin//CoNiS) prepared by different hot-pressing pressure (0, 0.2, 0.5 and 1.0 MPa) in 30 wt.% KOH at 60 ℃. (b) The cell voltage of MEAs with different hot-pressing pressure at various current density (500, 1000 and 2000 mA cm^{-2}). (c) Nyquist plot of MEAs with different hot-pressing pressure at 1.4 V_{cell} in 30 wt.% KOH at 60 ℃. (d) Polarization curves of C-MEA-CCS (CoNiS//X37-50//CoNiS) prepared by different hot-pressing pressure (0, 0.2, 0.5 and 1.0 MPa) in 30 wt.% KOH at 60 ℃. (e) The cell voltage of C-MEA-CCS

(CoNiS//X37-50//CoNiS) with different hot-pressing pressure at various current density (500, 1000 and 2000 mA cm^{-2}). (f) Nyquist plot of C-MEA-CCS (CoNiS//X37-50//CoNiS) with different hot-pressing pressure at 1.4 V_{cell} in 30 wt.% KOH at 60 °C. (g) Polarization curves of C-MEA-CCS (CoNiS//X37-50//CoNiS) prepared by different hot-pressing pressure (0, 0.2, 0.5 and 1.0 MPa) in 5 wt.% KOH at 60 °C. (e) The cell voltage of C-MEA-CCS (CoNiS//X37-50//CoNiS) with different hot-pressing pressure at various current density (250, 500 and 1000 mA cm^{-2}). (f) Nyquist plot of MEAs with different hot-pressing pressure at 1.4 V_{cell} in 5 wt.% KOH at 60 °C.

Supplementary Note 1

To further investigate the impact of the interfacial resistance on the performance of conventional MEA-CCS (C-MEA-CCS) using Zirfon membrane and self-supported electrodes, we utilized the hot-pressing method to regulate the binding force between self-supported electrodes and membranes. As shown in Supplementary Fig. 2a-b, the introduction of hot pressing will increase the electrolysis performance of MEAs, especially at high current densities. Furthermore, to characterize the ohmic resistances in MEAs, EIS was performed at 1.4 V (Supplementary Fig. 2c). The intercept in the high-frequency region represents the ohmic resistance, which consists of the ionic and electronic resistances. Improving the hot-pressing pressure reduced the ohmic resistance of cell.

We also investigated the impact of the interfacial resistance on the performance of C-MEA-CCS using Sustainion X37-50 membranes and self-supported electrodes. Supplementary Fig. 1 exhibited SEM imgaes of the AEM/eletrode interface after hot pressing under different pressures. It can be observed that the interface distance decreased as improving the hot-pressing pressure from 0 to 1.0 MPa. When 30 wt% KOH solution was used as the electrolyte, MEAs-CCS with different hot-pressing pressures showed discrepant electrolysis performance (Supplementary Fig.

2d). Typically, the MEA with 1.0 MPa showed cell voltage of 1.61, 1.76, and 2.03 V at different current densities of 500, 1000, and 2000 mA cm^{-2}, which were lower than that (1.67, 1.84, and 2.16 V) of the MEA with 0.1 MPa (Supplementary Fig. 2e). Furthermore, to characterize the ohmic resistances in MEAs, EIS was performed at 1.4 V (Supplementary Fig. 2f). Improving the hot-pressing pressure reduced the ohmic resistance of cell. This result indicates that reducing the interfacial resistance between the electrode and the membrane can obviously improve the electrolysis performance. When 10 wt% KOH solution was used as the electrolyte, the electrolysis performance difference of MEAs-CCS with different hot-pressing pressures was significant (Supplementary Fig. 2g). Typically, the MEA with 1.0 MPa showed cell voltage of 1.54, 1.68 and 1.92 V at different current density of 250, 500 and 1000 mA cm^{-2}, which are significantly lower than that (1.65, 1.87 and 2.3 V) of the MEA with 0.1 MPa (Supplementary Fig. 2h). Improving the hot-pressing pressure can significantly reduce the ohmic resistance of cells, especially at low-concentration KOH solution (Supplementary Fig. 2i).

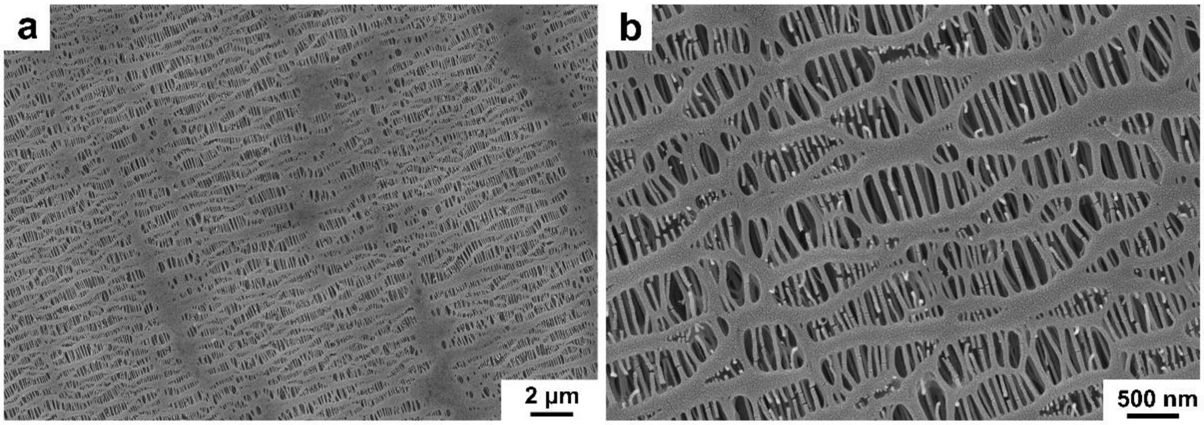

Supplementary Fig. 3 | Scanning electron microscopy (SEM) analysis of polypropylene (PP) membrane. (a-b) The SEM images of pristine PP porous membranes.

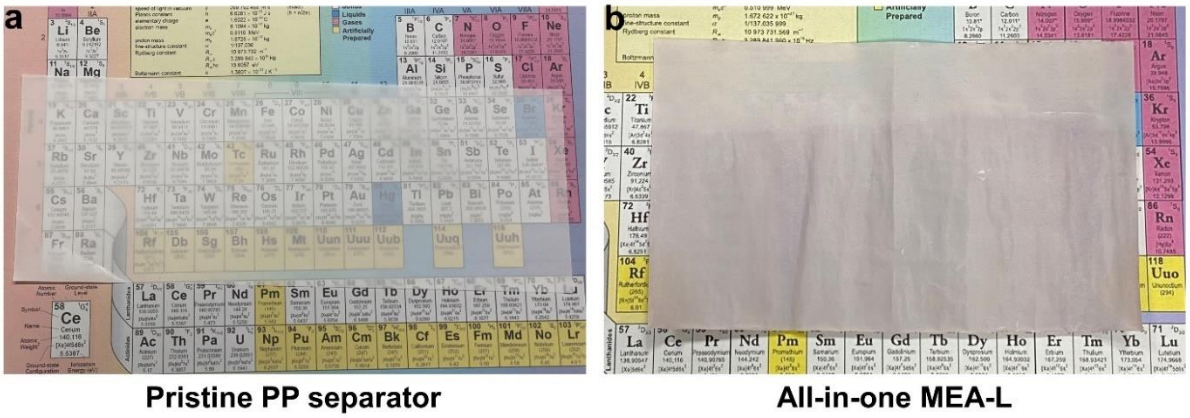

Supplementary Fig. 4 | Photographic images of PP separator and all-in-one MEA-L. Photographs of (a) pristine PP separator (size: 40 cm × 20 cm; thickness: ~20 cm) and (b) all-in-one MEA-L (size: 40 cm × 20 cm; thickness: ~26 cm).

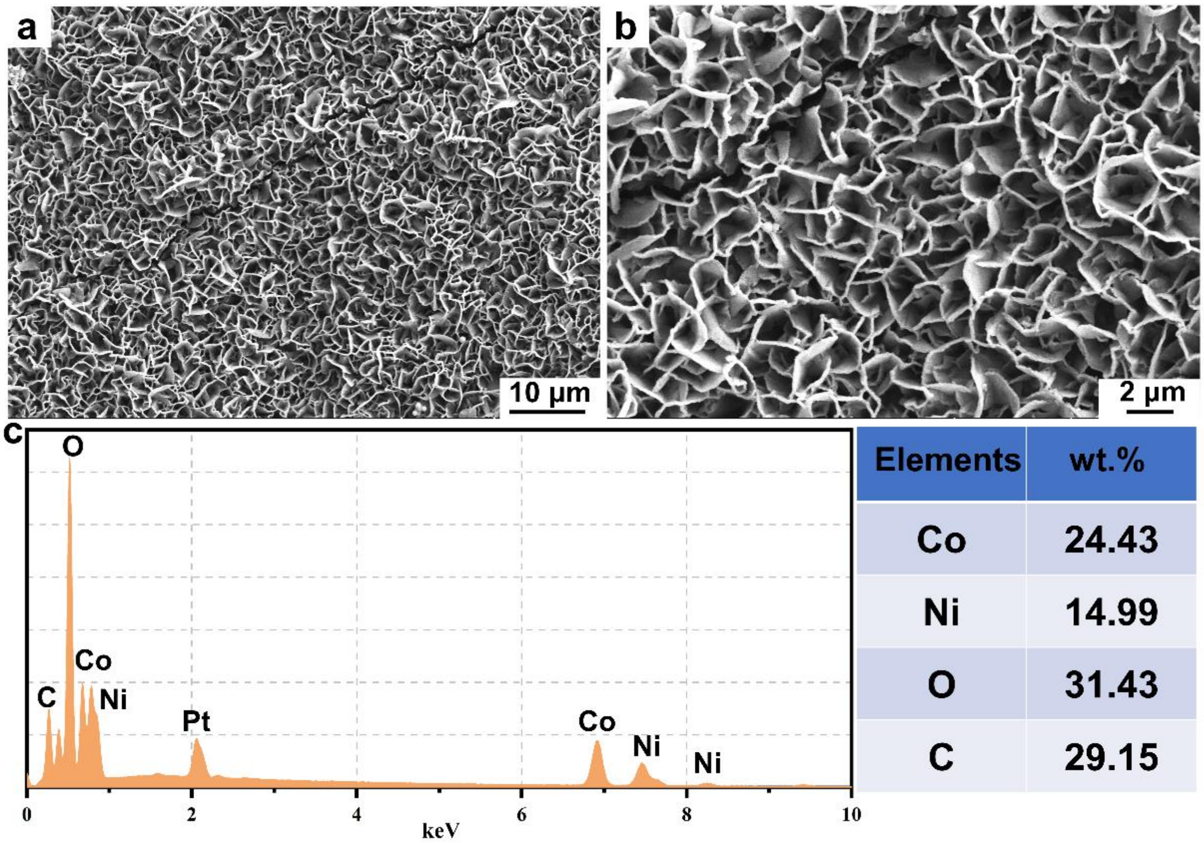

Supplementary Fig. 5 | SEM analysis of all-in-one MEA-L. SEM images of (a-b) catalyst layer morphologyies of all-in-one MEA-L. (c) The EDS spectrum of the selected area in catalyst layer surface of all-in-one MEA-L.

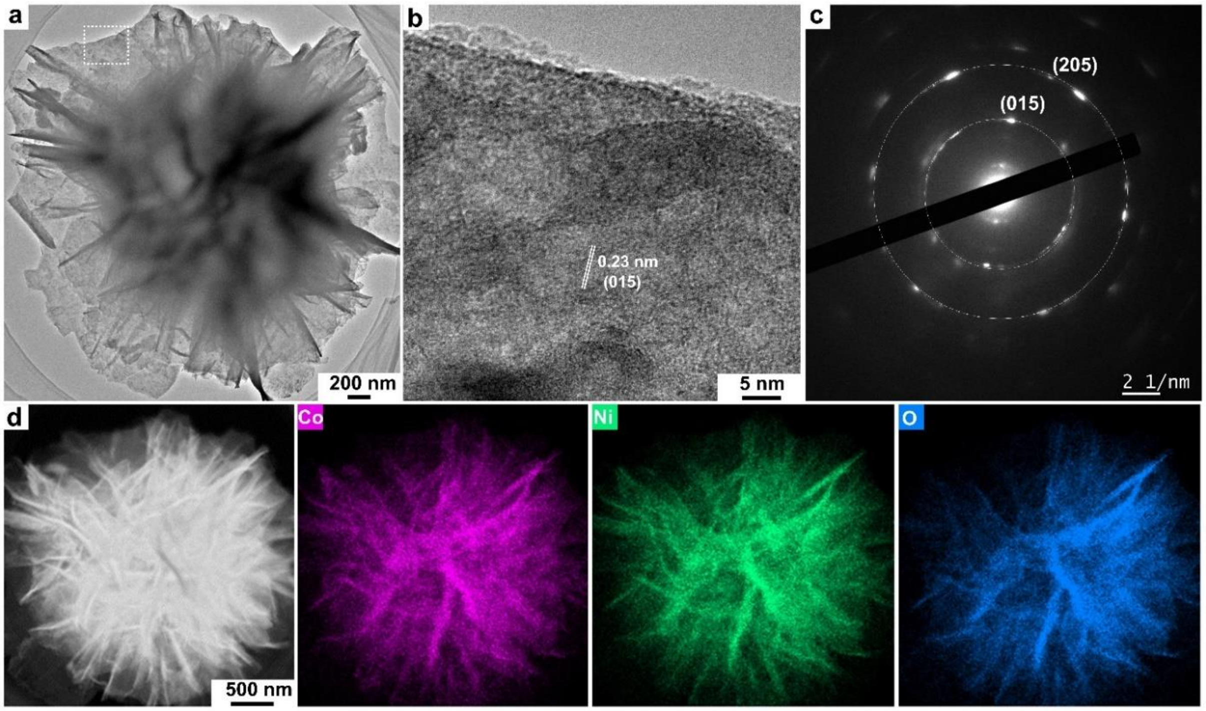

Supplementary Fig. 6 | Structure characterization of CoNi LDH by transmission electron microscope (TEM). (a) TEM and (b) high-resolution TEM images of CoNi LDH. (c) SAED pattern of CoNi LDH. (d) The corresponding elemental mapping images of Co, Ni and O in CoNi LDH.

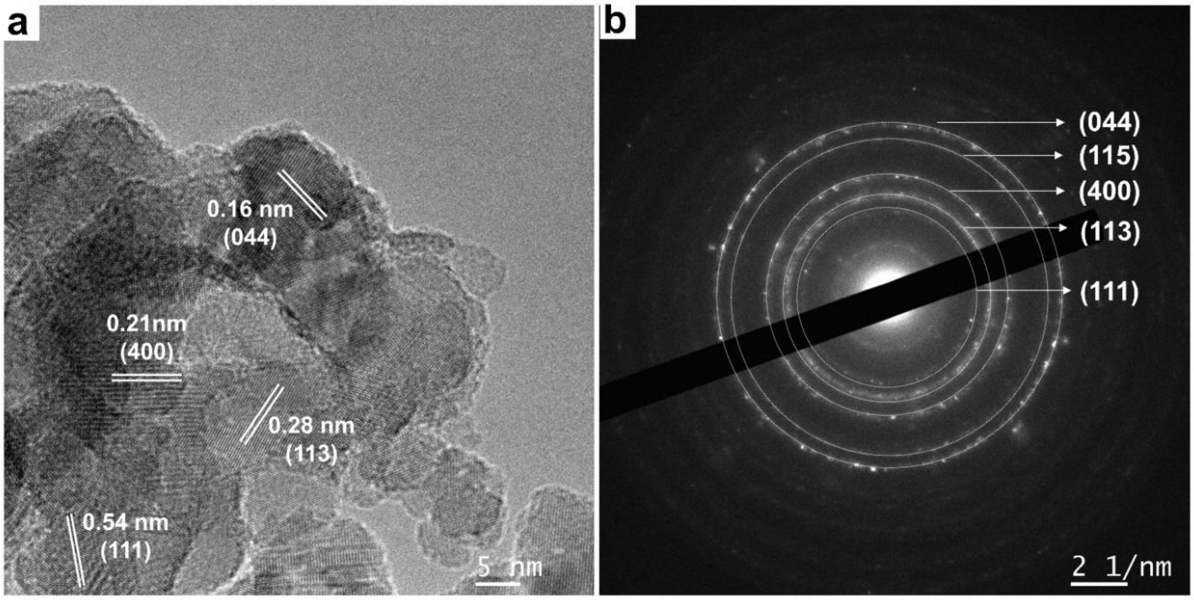

Supplementary Fig. 7 | Structure characterization of CoNiS by TEM. (a) The high-resolution TEM image of CoNiS nanosheets, and (b) the SAED pattern of the area in (a).

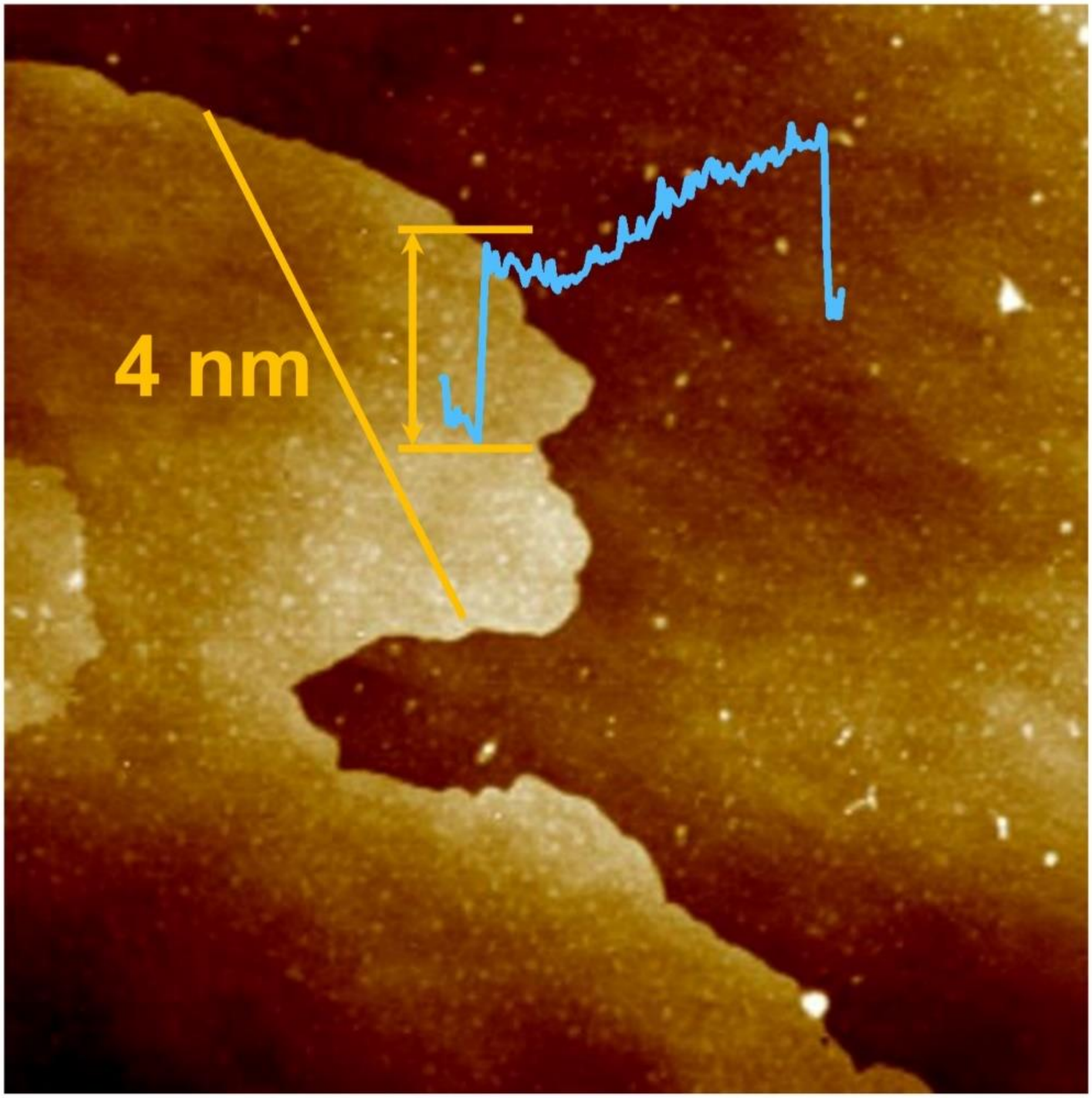

Supplementary Fig. 8 | AFM characterization of CoNiS nanosheets. AFM image of CoNiS nanosheets and corresponding height profile along the marked yellow line.

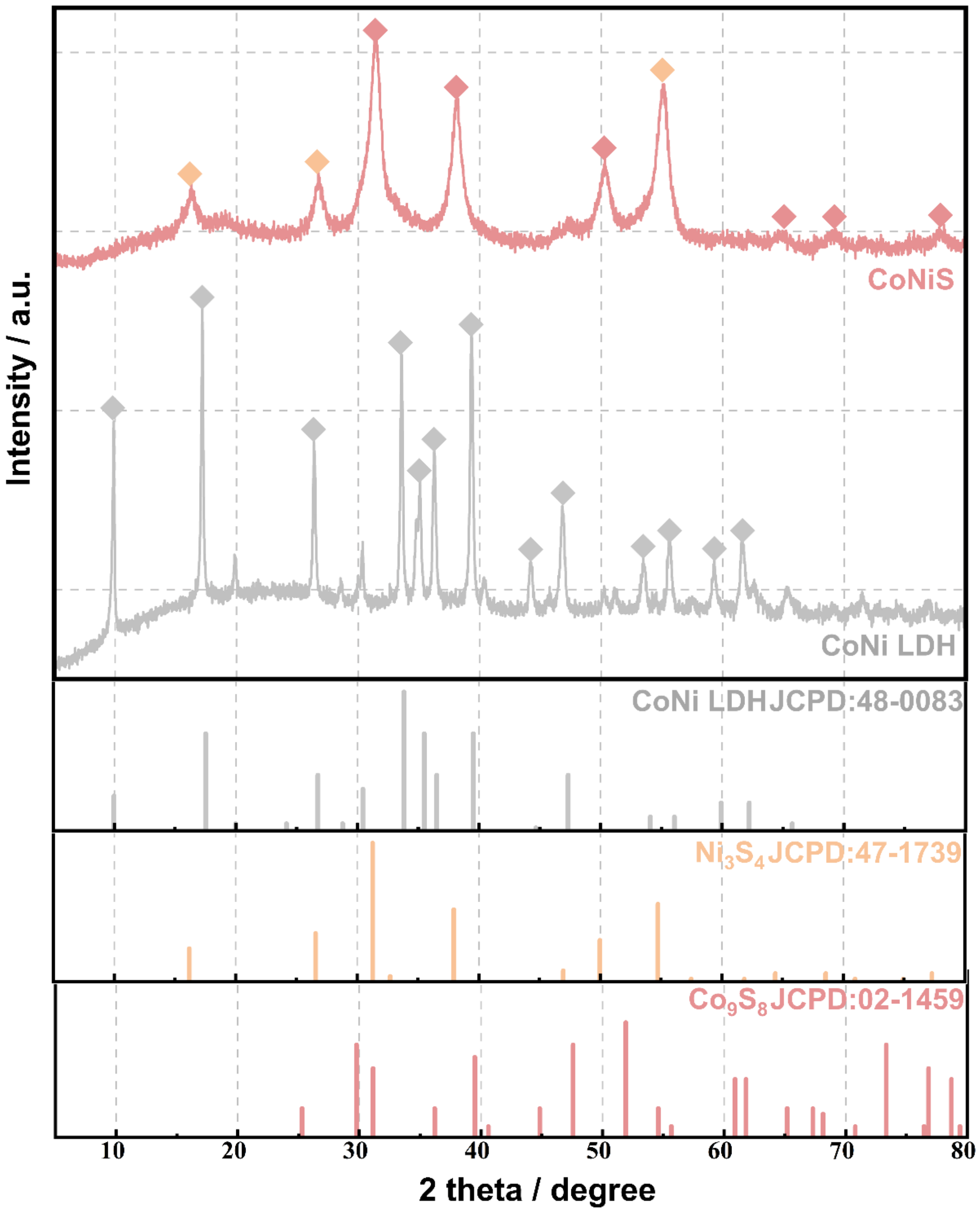

Supplementary Fig. 9 | XRD patterns. XRD patterns of CLs of all-in-one MEA-L and all-in-one MEA-S.

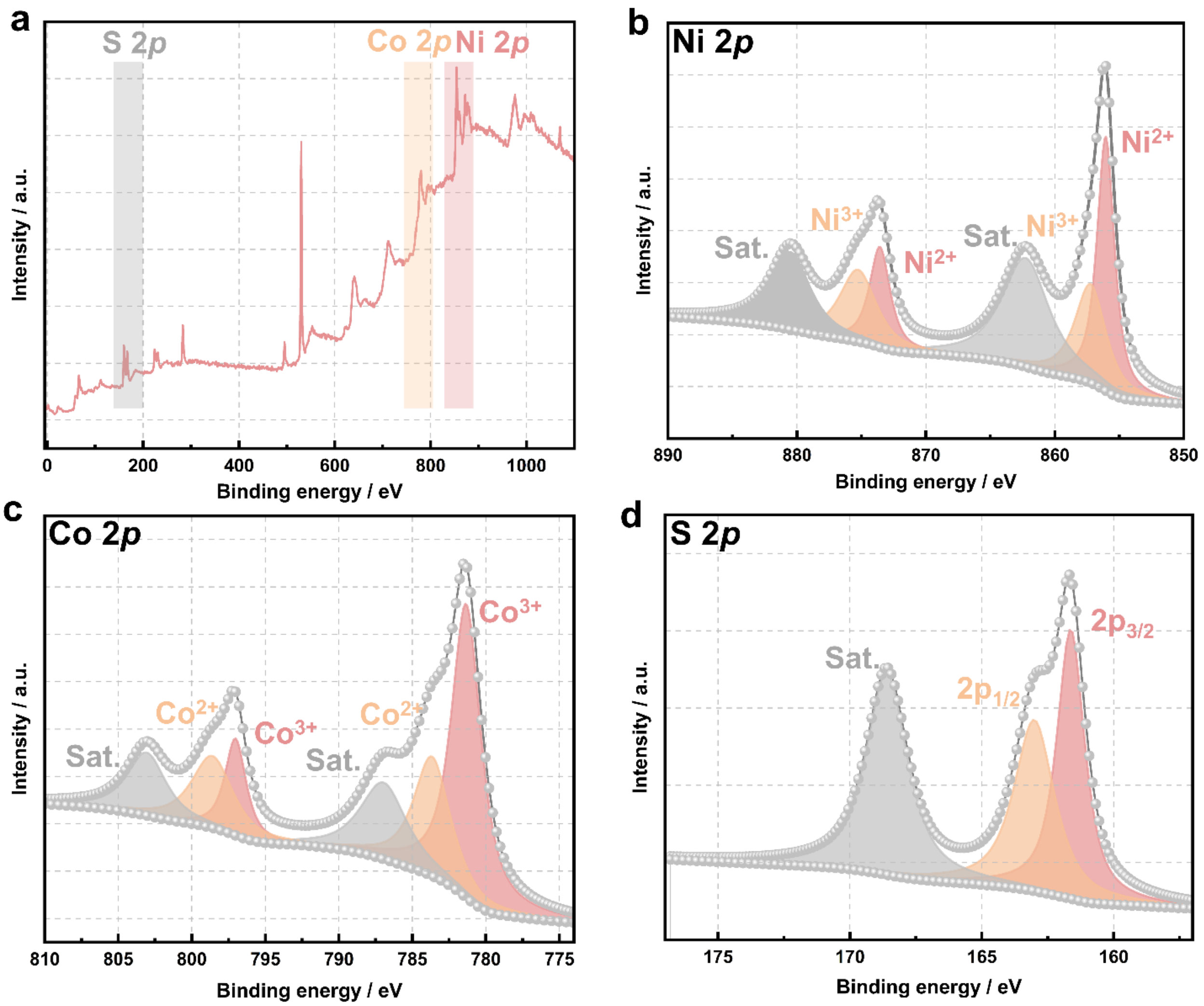

Supplementary Fig. 10 | XPS spectra. (a) XPS survey of CoNiS. High-resolution XPS spectra of (b) Ni 2*p* for CoNiS. High-resolution XPS spectra of (c) Co 2*p* for CoNiS. High-resolution XPS spectra of (d) S 2*p* for CoNiS.

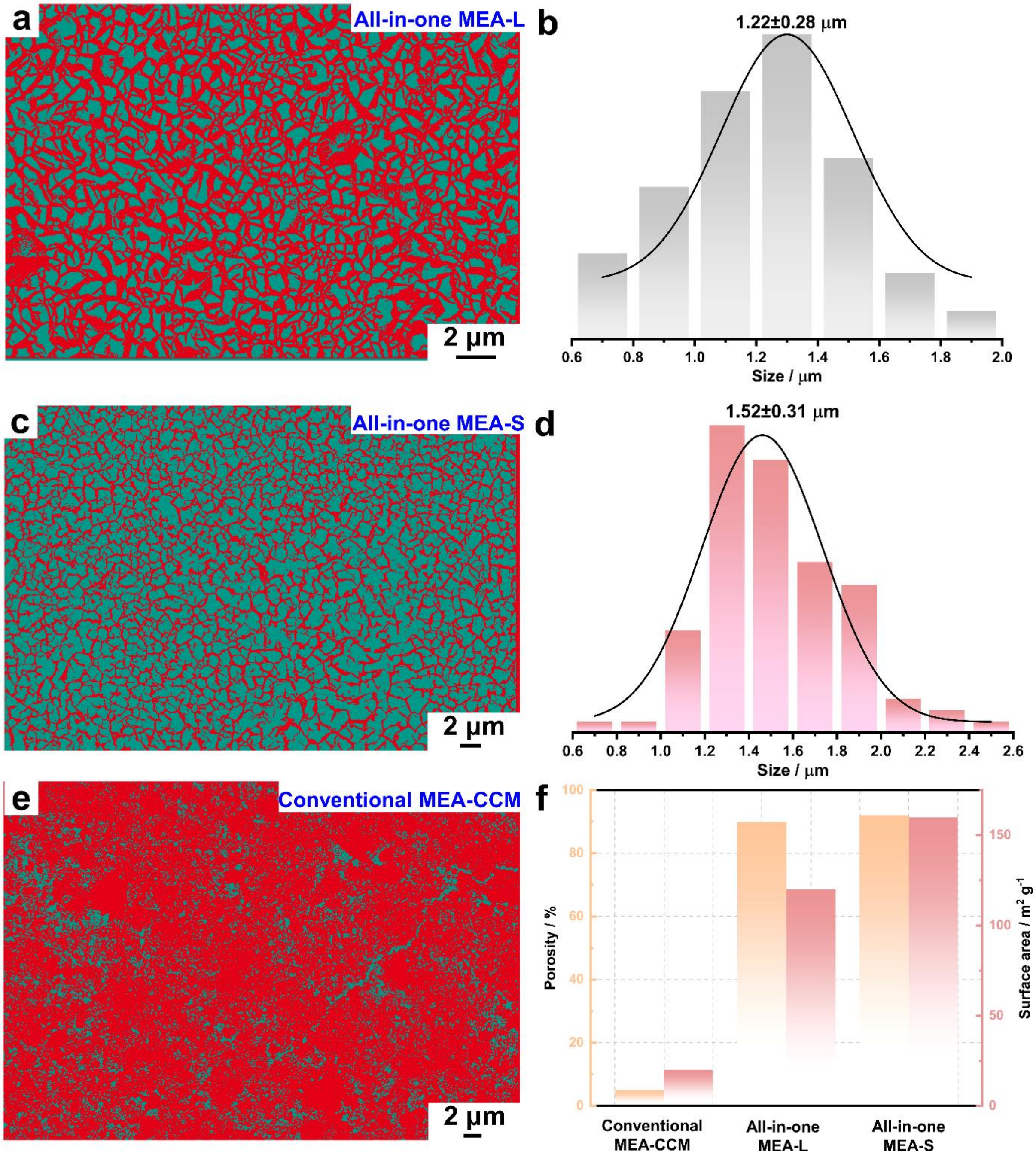

Supplementary Fig. 11 | The CL images for poresity and pore structures analysis. (a) all-in-one MEA-L, (c) all-in-one MEA-S and (e) conventional MEA-CCM (The green parts are pores; The red parts are solid catalysts). The pore size distribution of (b) all-in-one MEA-L and (d) all-in-one MEA-S. (f) The porosity and surface area of conventional MEA-CCM, all-in-one MEA-L and all-in-one MEA-S. Image analysis, i.e. the extraction of areal surface porosity (ASP), was performed using the Fiji distribution of the ImageJ software program. A Gaussian blur was applied to pristine SEM images to reduce the roughness of

the exterior surfaces and filter out small, illuminated intraporous surfaces while maintaining image integrity. A threshold was applied using the default method of the ImageJ threshold operation, then the image was inverted.

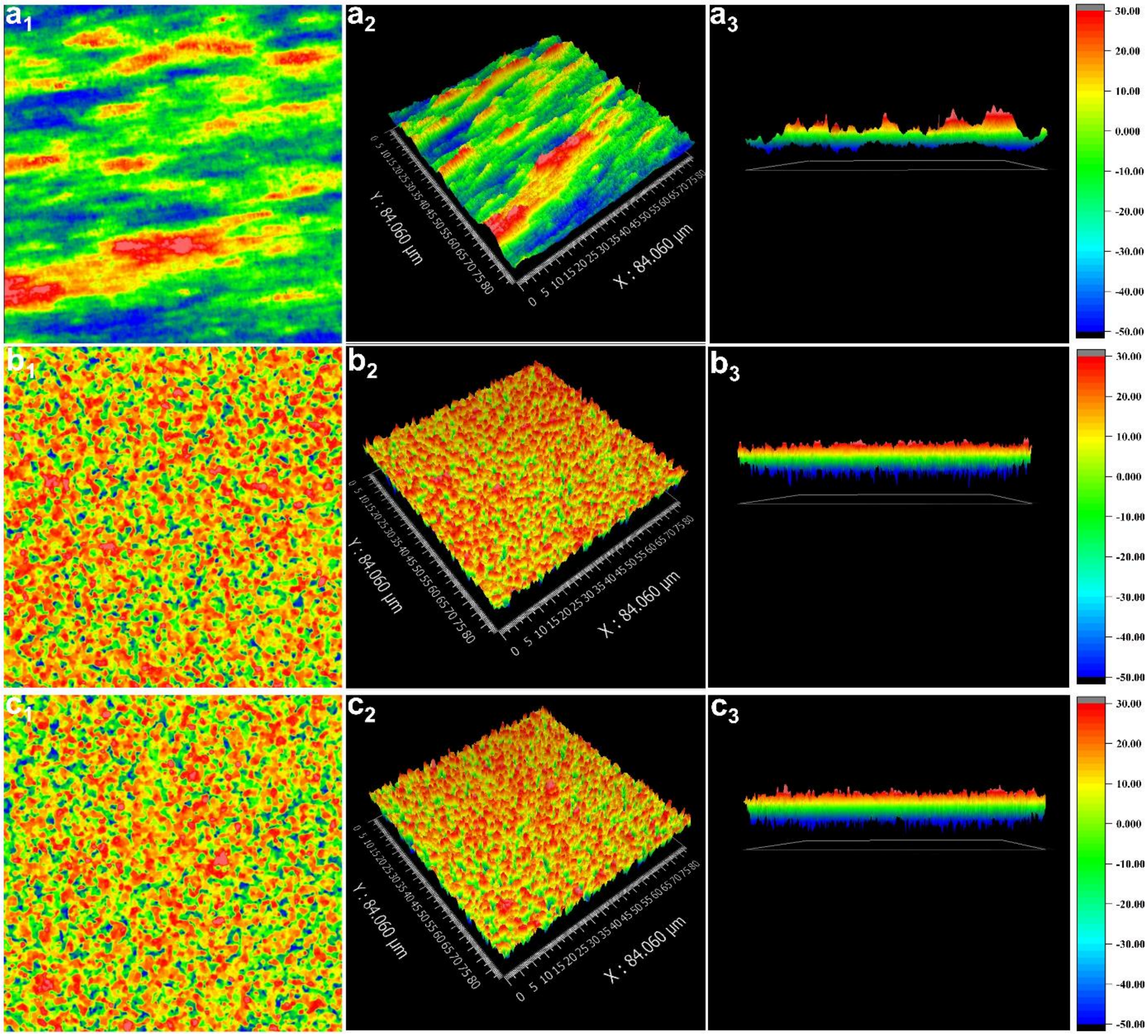

Supplementary Fig. 12 | White light interferometry characterization of PP membrane and CLs structure of all-in-one MEAs. (a_1) 2D and (a_2-a_3) 3D images of pristine PP porous membrane. (b_1) 2D and (b_2-b_3) 3D images of all-in-one MEA-L. (c_1) 2D and (c_2-c_3) 3D images of all-in-one MEA-S.

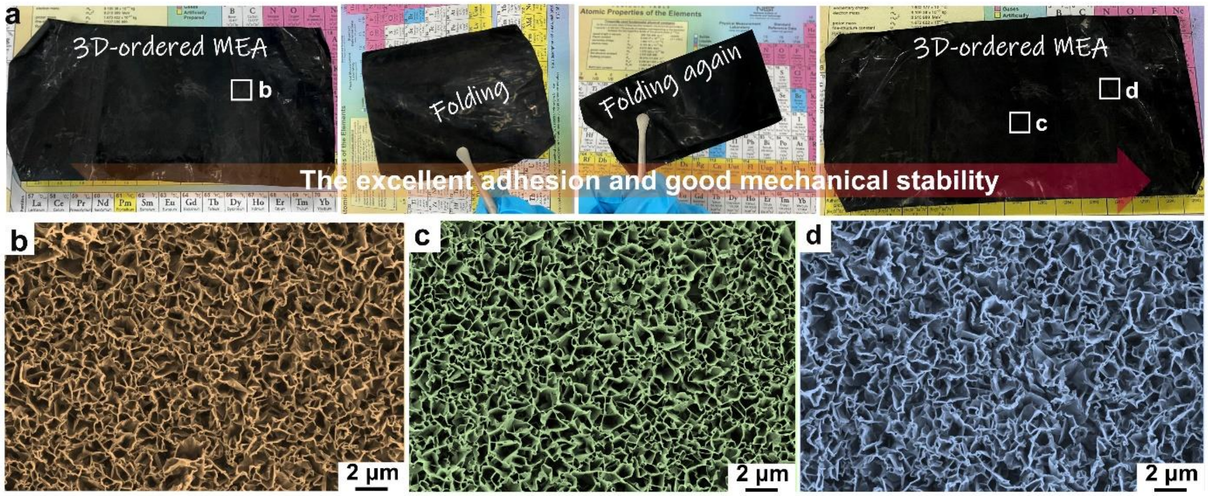

Supplementary Fig. 13 | Mechanical toughness and catalyst layer adhesion tests of all-in-one MEA-S. (a) The all-in-one MEA-S (size: 40 cm × 20 cm; thickness: ~26 cm) recovers the shape without cracks even after the twisting and kneading tests. The corresponding SEM images of the representative areas (b), (c) and (d), respectively.

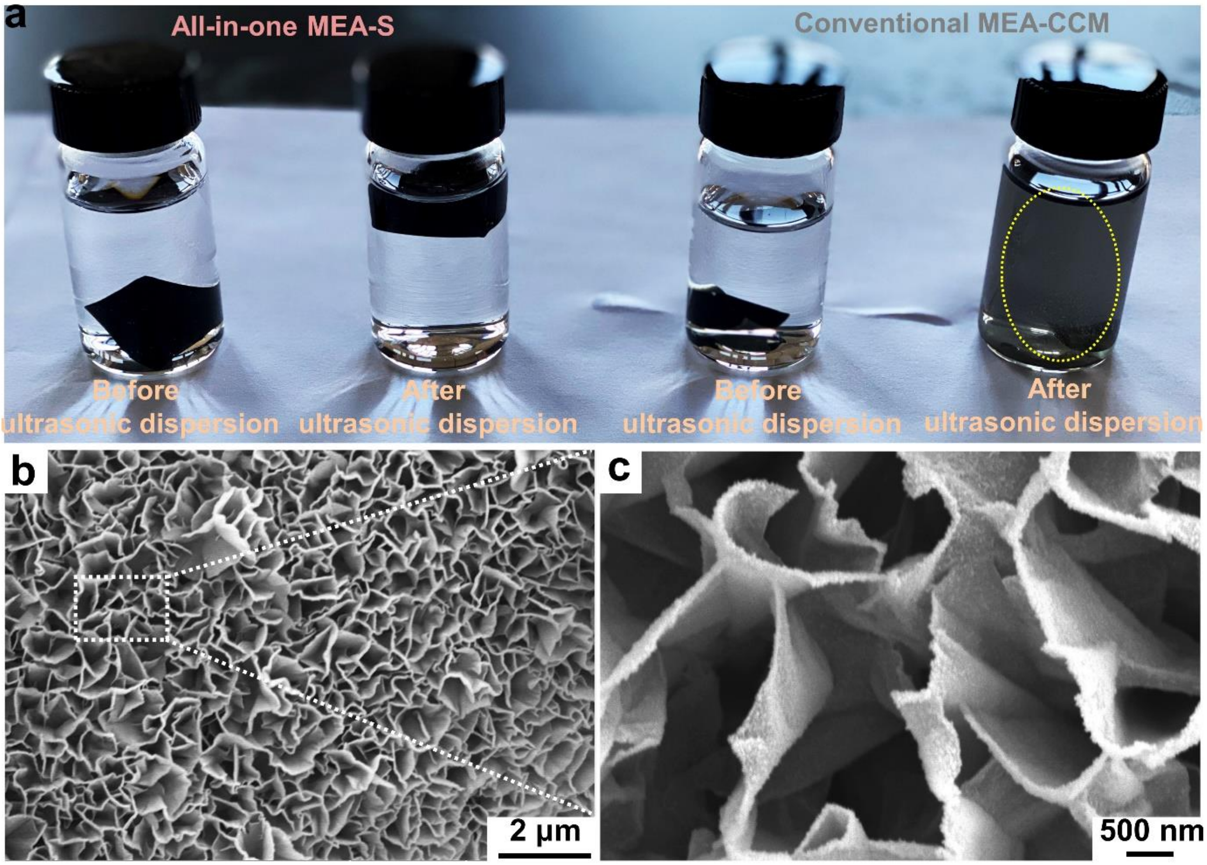

Supplementary Fig. 14 | Catalyst layer adhesion tests using ultrasonic vibration method. (a) The digital photographs of all-in-one MEA-S before and after ultrasonication for 10 min. (b-c) SEM images of catalyst layers of all-in-one MEA-S after ultrasonication for 10 min.

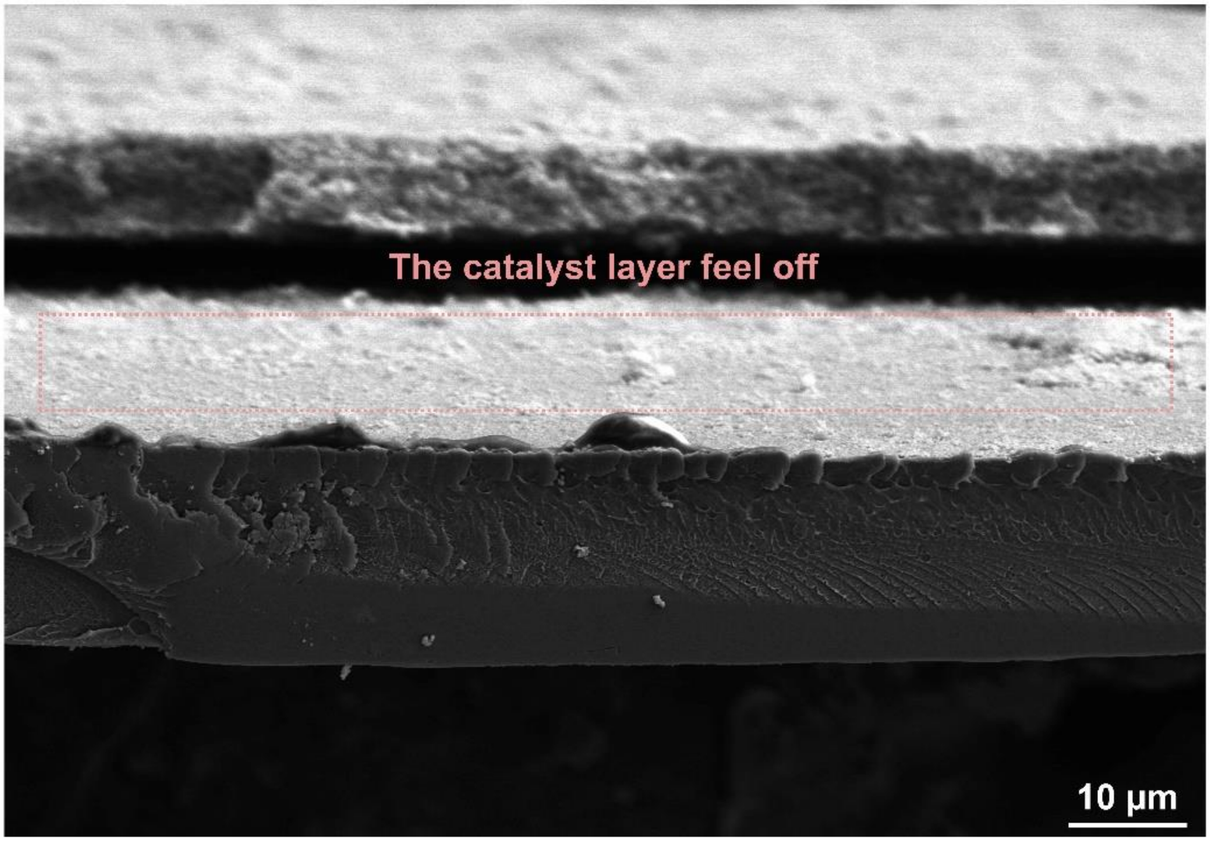

Supplementary Fig. 15 | SEM analysis of conventional MEA-CCM (C-MEA-CCM). SEM images of catalyst layer of the C-MEA-CCM after ultrasonication.

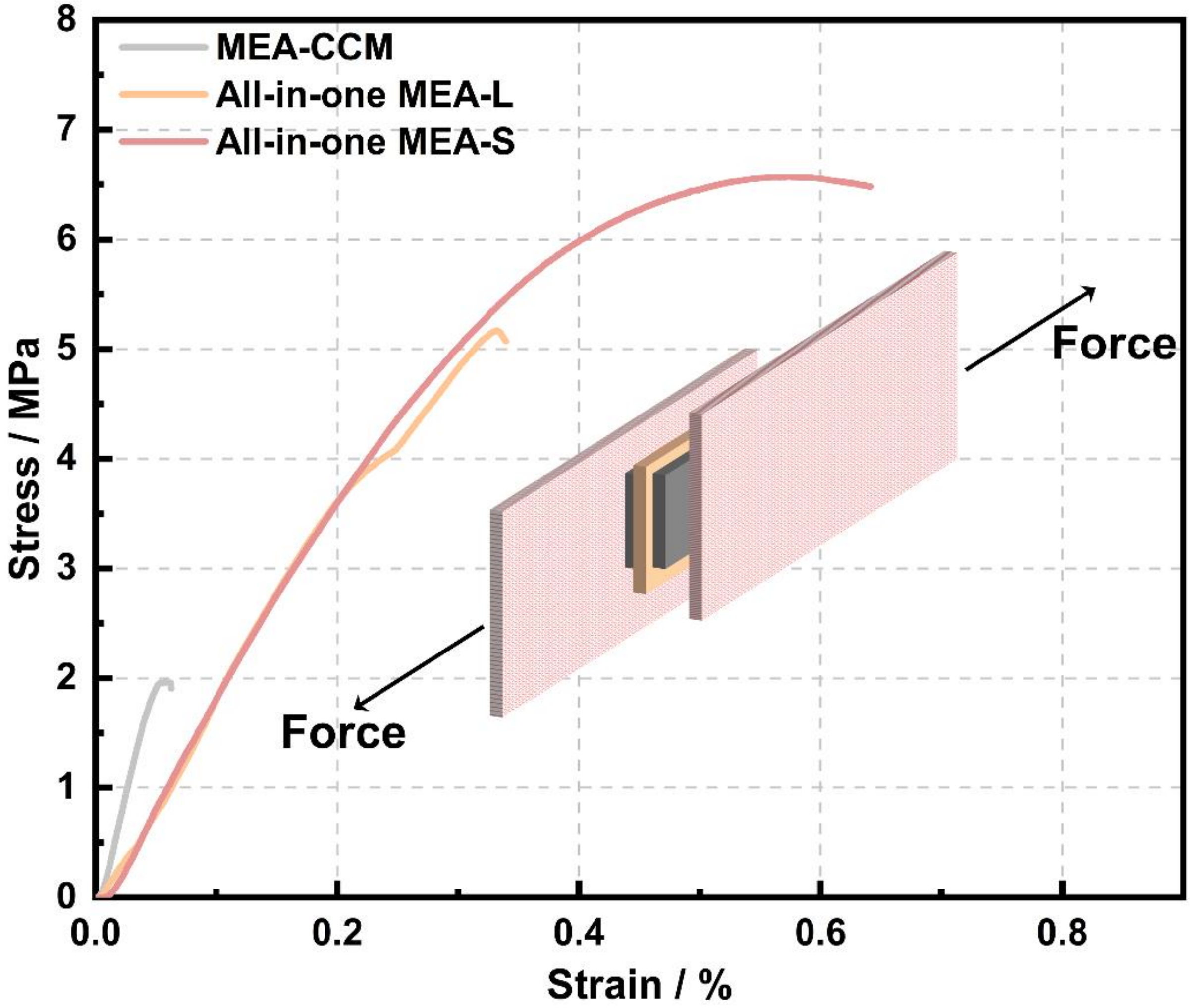

Supplementary Fig. 16 | Catalyst layer adhesion tests. Tensile stress–strain curves for shear testing of the dry conventional MEA-CCM, the dry all-in-one MEA-L, and the all-in-one MEA-S.

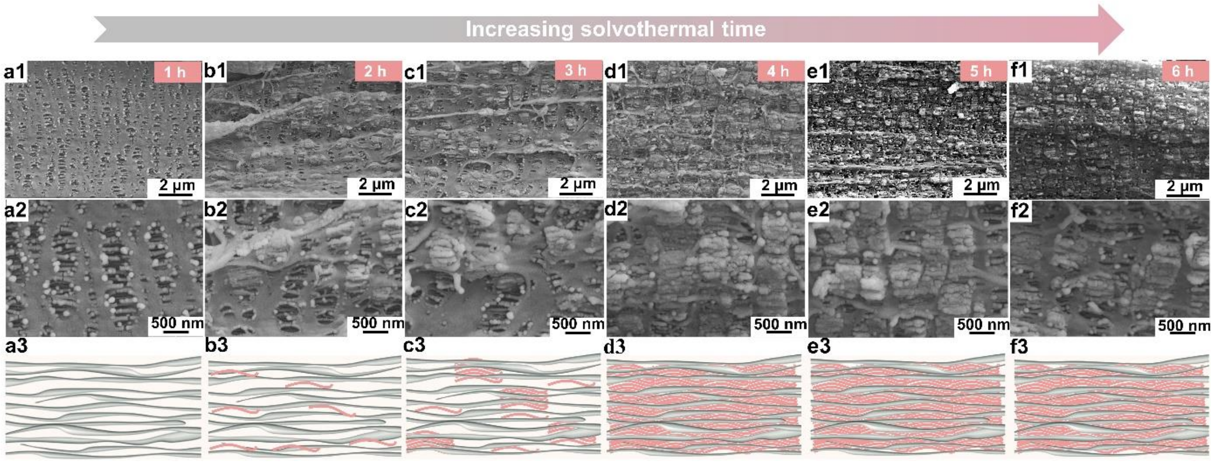

Supplementary Fig. 17 | SEM analysis of cross-sectional morphologies of all-in-one MEA-S with different solvothermal time. (a1-a2) 1 h, (b1-b2) 2 h, (c1-c2) 3 h, (d1-d2) 4 h, (e1-e2) 5 h and (f1-f2) 6 h.

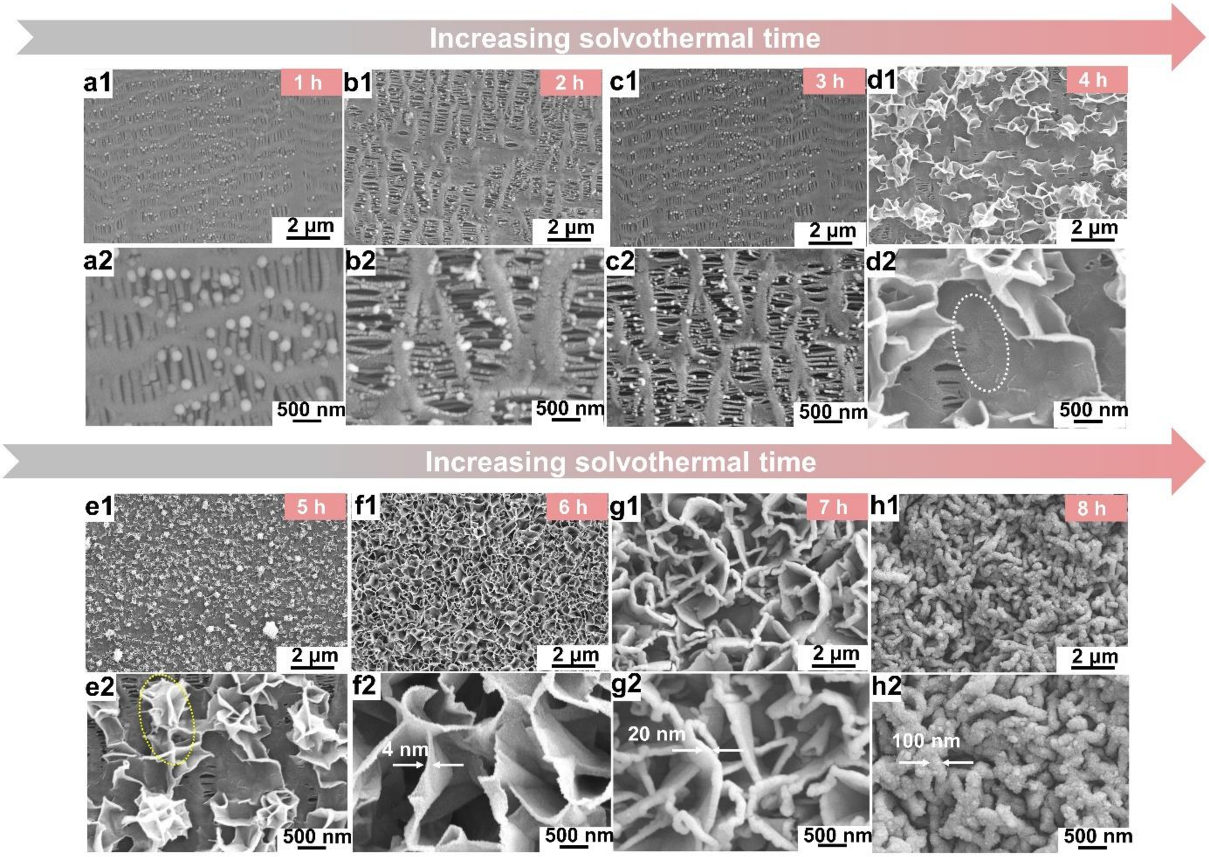

Supplementary Fig. 18 | SEM analysis of catalyst layer morphologies of all-in-one MEA-S with different solvothermal time. (a1-a2) 1 h, (b1-b2) 2 h, (c1-c2) 3 h, (d1-d2) 4 h, (e1-e2) 5 h, (f1-f2) 6 h, (g1-g2) 7 h and (h1-h2) 8 h.

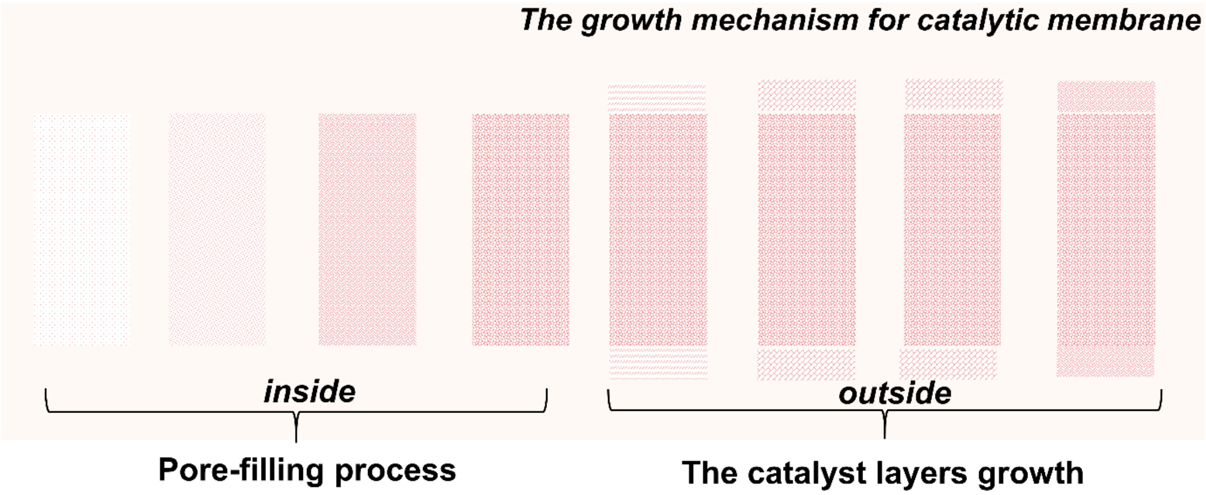

Supplementary Fig. 19 | Schematic diagrams of the growth mechanism for catalytic membrane. The oriented intergrowth process of catalyst layer in MEA can be divided into two stages: the pore-filling process and the catalyst layer grwoth on membrane.

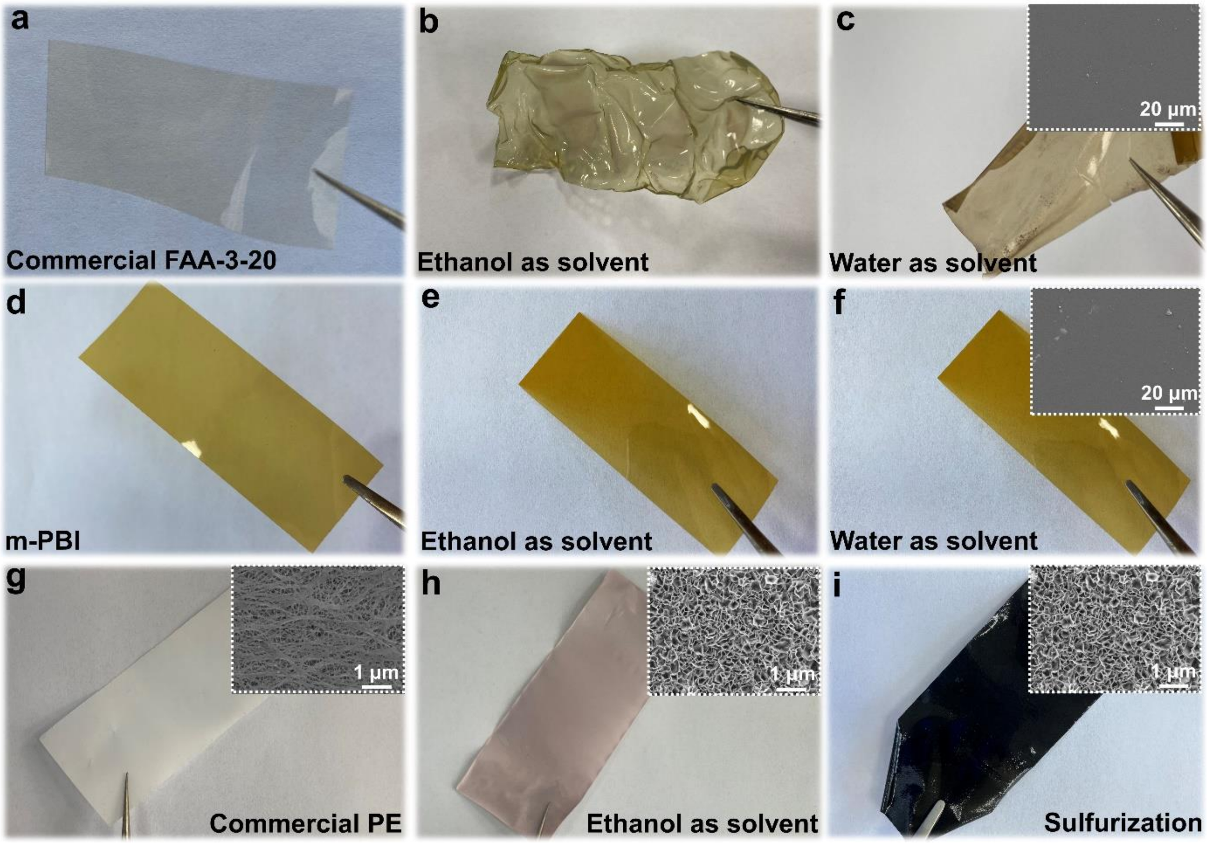

Supplementary Fig. 20 | Photographic images of different membranes before and after CL intergrowth process. The digital photographs of (a) pristine commercial FAA-3-20 membrane, (b) commercial FAA-3-20 membrane after solvothermal reaction (ethanol as solvent), (c) commercail FAA-3-20 membrane after hydrothermal reaction (water as solvent), (d) pristine PBI membrane, (e) PBI membrane after solvothermal reaction (ethanol as solvent), (f) PBI membrane after hydrothermal reaction (water as solvent), (g) commercial PE membrane, (h) commercial PE membrane after solvothermal reaction (ethanol as solvent) and (i) commercail PE membrane after sulfurization treatment.

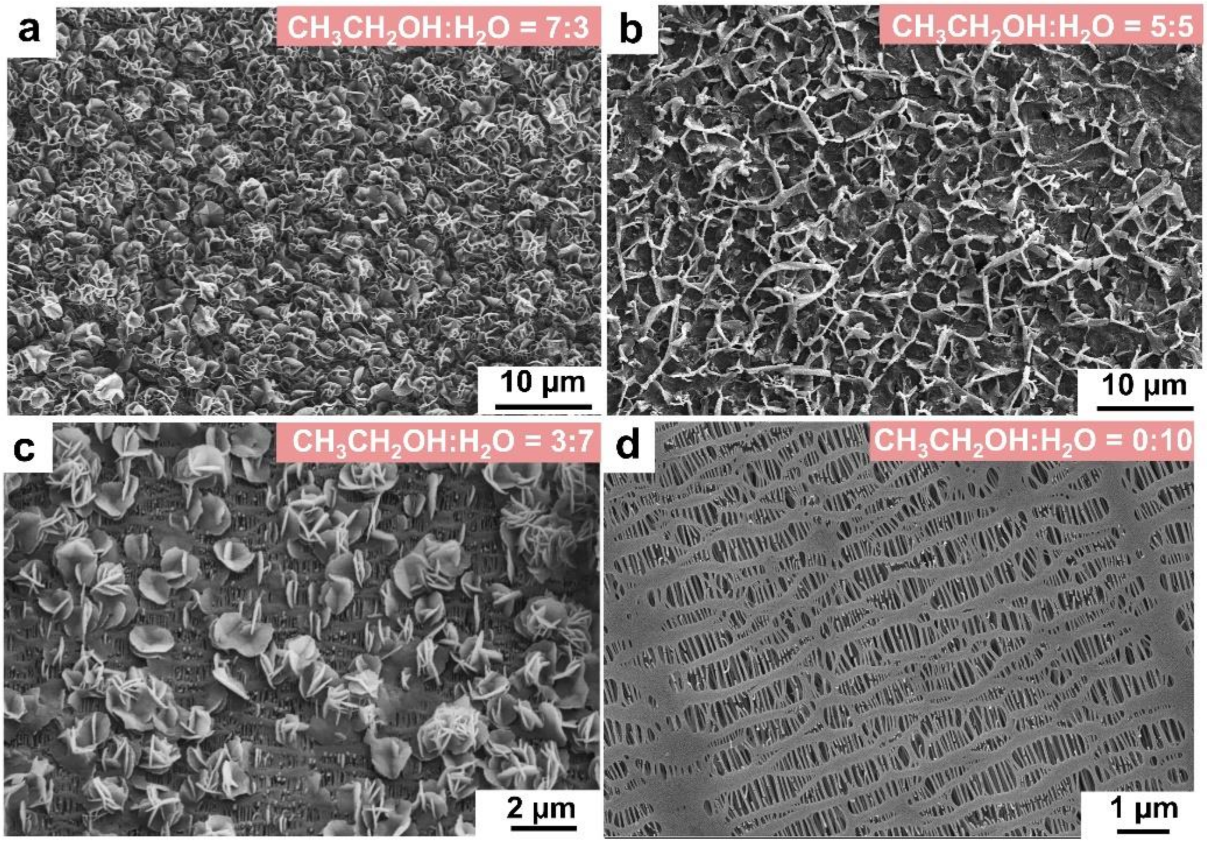

Supplementary Fig. 21 | SEM analysis of all-in-one MEA-S. SEM imgaes of catslyst layer surface morphologies of all-in-one MEA-S with different ratio of ethanol and water: (a) ethanol : H_2O = 7:3, (b) ethanol : H_2O = 5:5, (c) ethanol : H_2O = 3:7 and (d) ethanol : H_2O = 0:10.

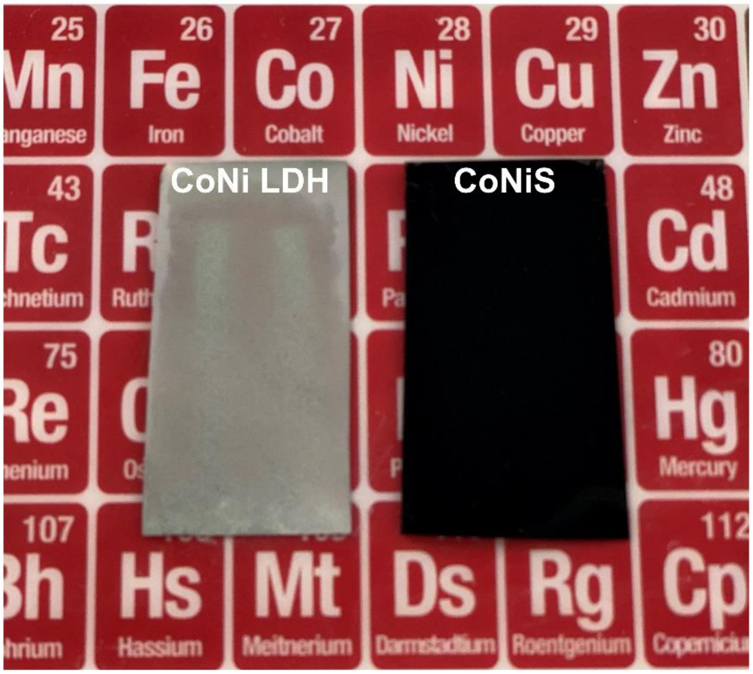

Supplementary Fig. 22 | Photographic images of CoNi LDH/Ni foil and CoNiS/Ni foil.

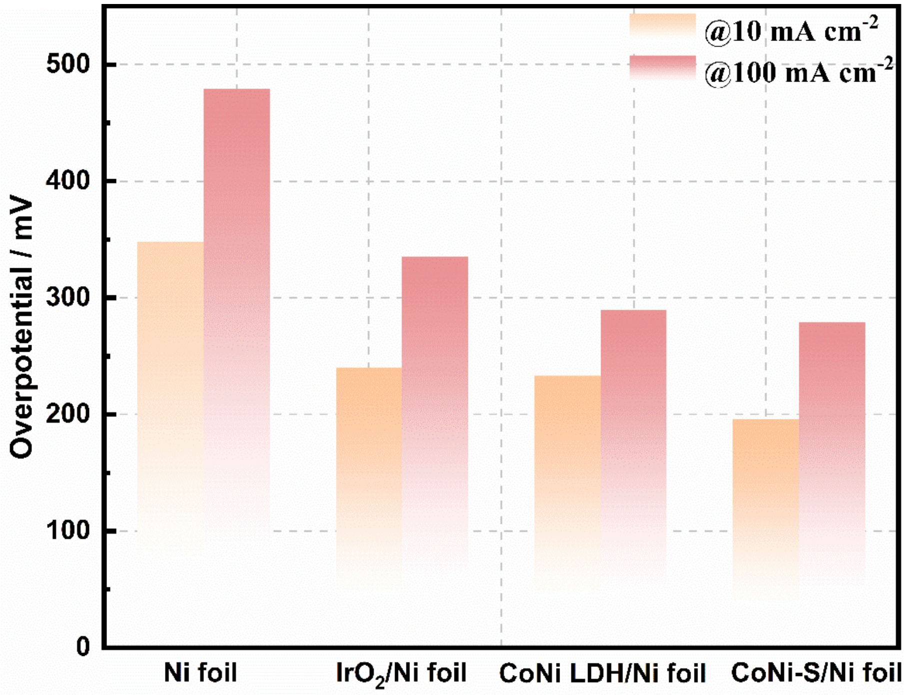

Supplementary Fig. 23 | OER overpotential analysis of catalysts at different current densities. The overpotentials of Ni foil, commercial IrO_2/Ni foil, CoNi LDH/Ni foil and CoNiS/Ni foil at 10 and 100 mA cm^{-2} towards OER.

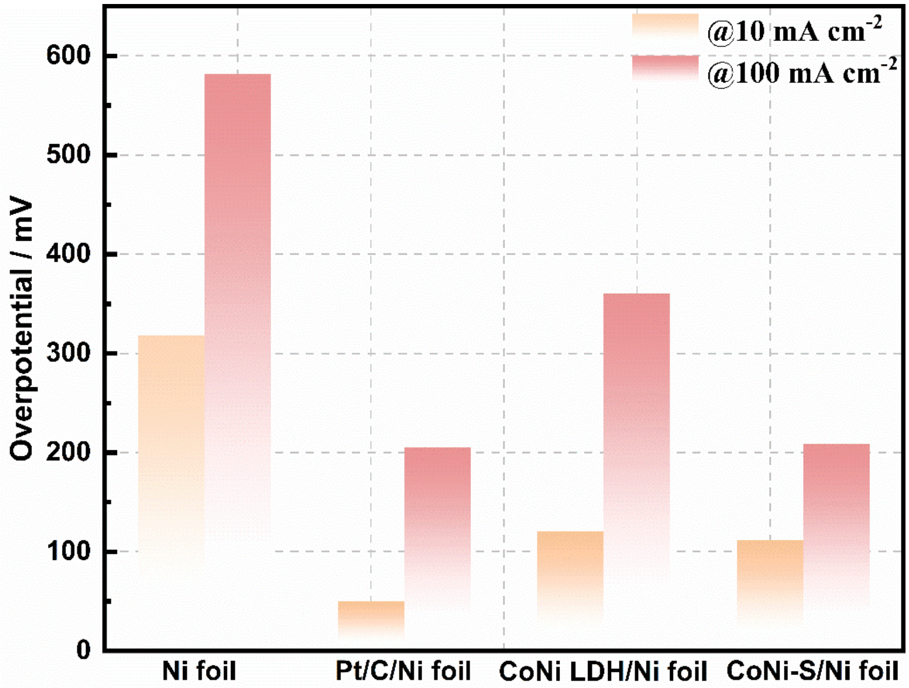

Supplementary Fig. 24 | HER overpotential analysis of catalysts at different current densities. The overpotential of Ni foil, commercial Pt/C/Ni foil, CoNi LDH/Ni foil and CoNiS/Ni foil at 10 and 100 mA cm^{-2} towards HER.

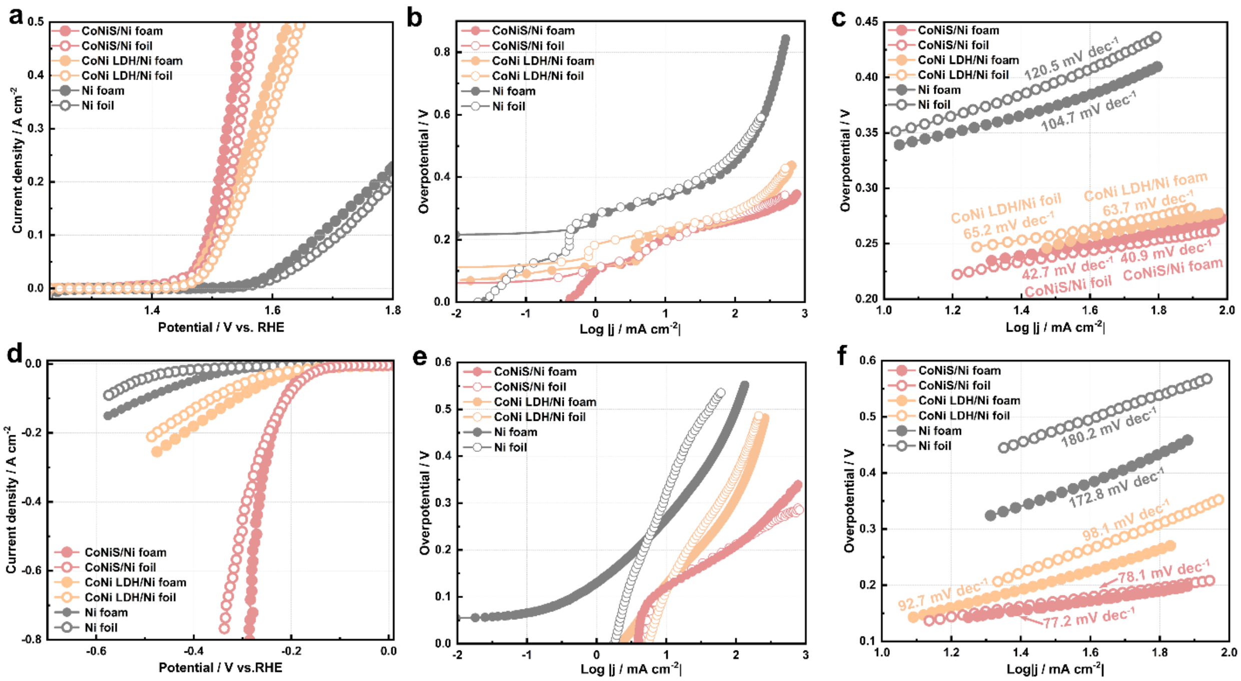

Supplementary Fig. 25 | Electrocatalytic properties for OER and HER. (a) OER polarization curves of CoNiS/Ni foam, CoNiS/Ni foil, CoNi LDH/Ni foam, CoNi/Ni foil, Ni foam and Ni foil in 1.0 M KOH at a scan rate of 5 mV s^{-1}. (b-c) The corresponding Tafel plots. (d) HER polarization curves of CoNiS/Ni foam, CoNiS/Ni foil, CoNi LDH/Ni foam, CoNi/Ni foil, Ni foam and Ni foil in 1.0 M KOH at a scan rate of 5 mV s^{-1}. (e-f) The corresponding Tafel plots.

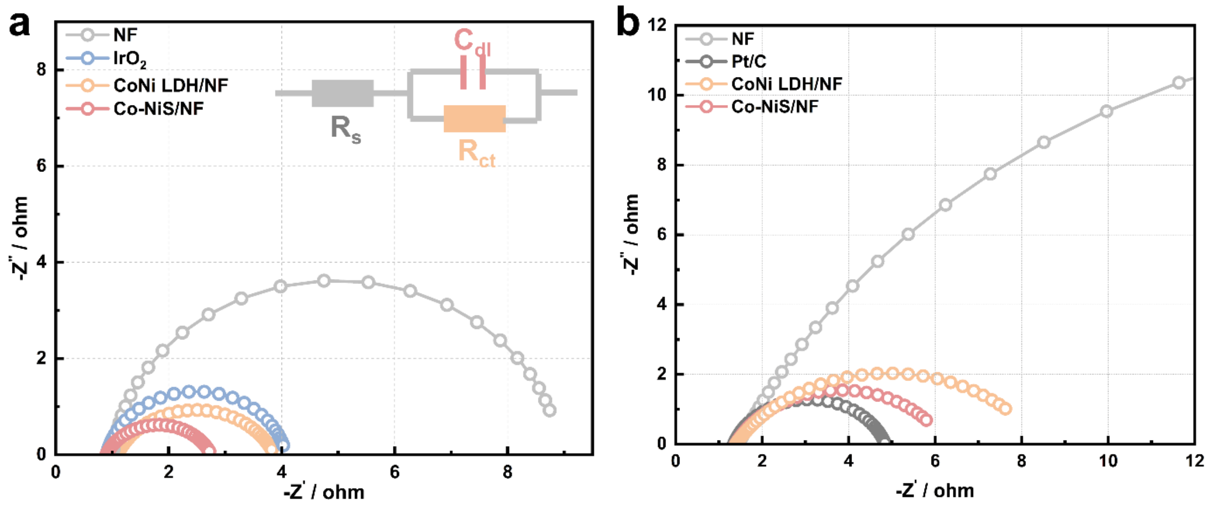

Supplementary Fig. 26 | EIS analysis of catalysts. (a) Nyquist plots of Ni foil, commercial IrO_2/Ni foil, CoNi LDH/Ni foil and CoNiS/Ni foil for OER. (b) Nyquist plots of Ni foil, commercial Pt/C/Ni foil, CoNi LDH/Ni foil and CoNiS/Ni foil for HER.

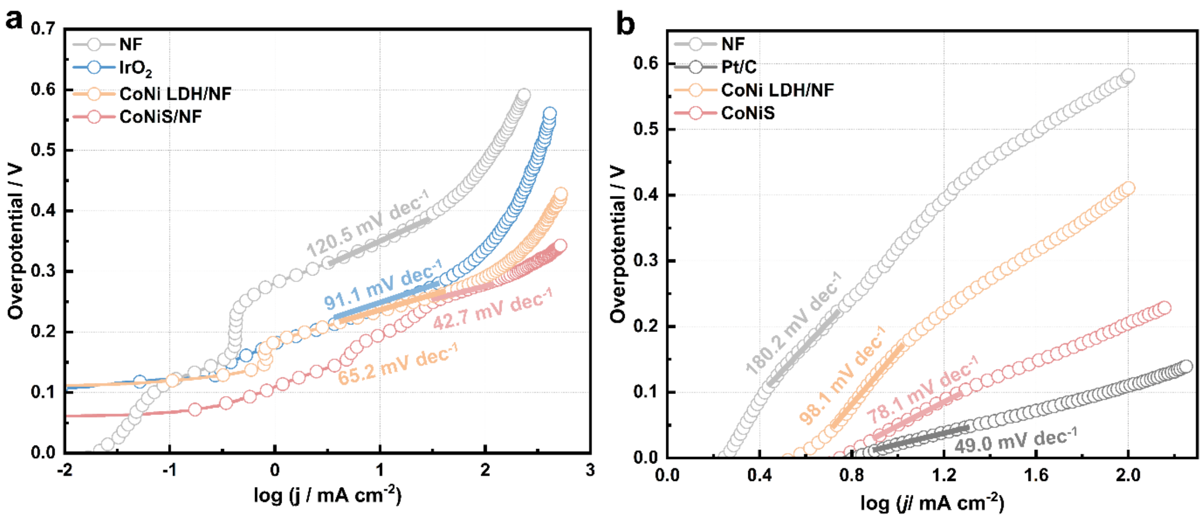

Supplementary Fig. 27 | Tafel slope analysis of catalysts. (a) Tafel plots for OER over Ni foil, commercial IrO_2/Ni foil, CoNi LDH/Ni foil and CoNiS/Ni foil. (b) Tafel plots for HER over Ni foil, commercial Pt/C/Ni foil, CoNi LDH/Ni foil and CoNiS/Ni foil.

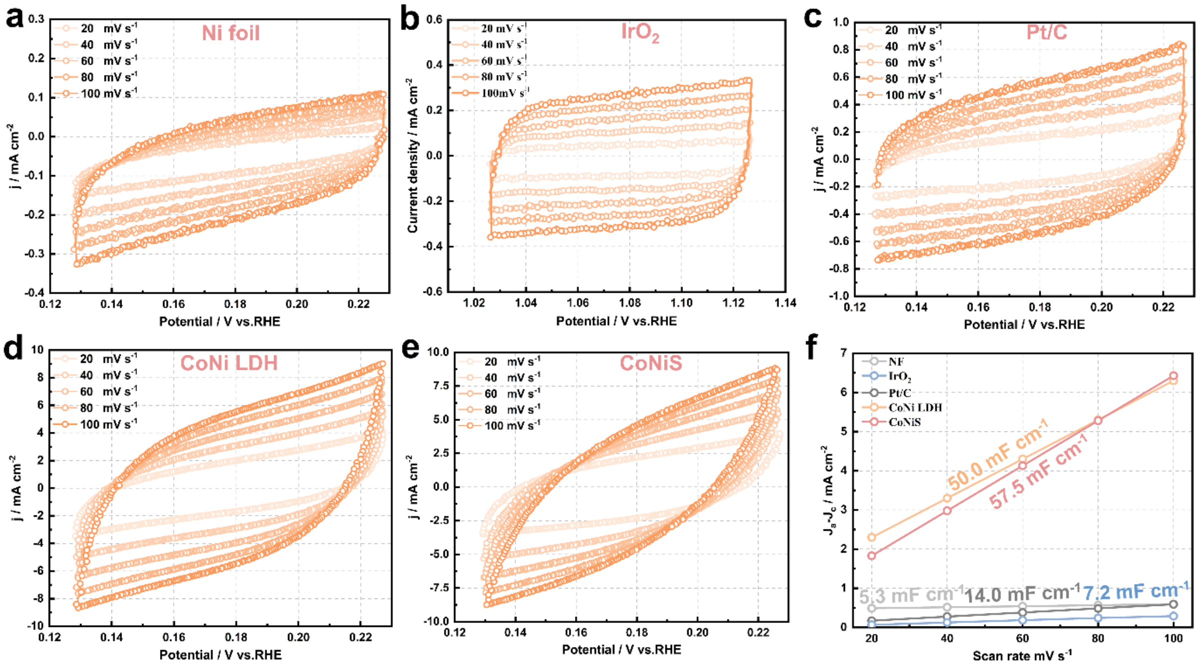

Supplementary Fig. 28 | Electrochemically active surface area (ECSA) calculation. Cyclic voltammetry (CV) curves of (a) Ni foil, (b) IrO_2/Ni foil, (c) Pt/C, (d) CoNi LDH/Ni foil and (e) CoNiS/Ni foil with different scan rates (20−100 mV s^{-1}). (f) Contrastive C_{dl} of the catalysts obtained from CV curves with different scanning rates.

Supplementary Note 2

To measure the electrochemical double layer capacitance of the samples, the current-potential curves were tested in a non-Faradaic HER or OER region of the voltammogram at speed rate of 20~100 mV s^{-1}. The double layer charging current i, normalized on the geometric area of the electrode, is directly proportional to the scan rate v ($i=vC_{dl}$). The double-layer capacitance (C_{dl}) was derived as the linear fitted slope by plotting the current against the scan rate. The ECSA of the catalyst layer can be calculated (ECSA= C_{dl}/C_s). Here, the specific capacitances (C_s) were chosen as C_s= 0.04 mF cm^{-2} based on typical reported values.

Ni foil:

$$A_{ECSA}^{NF} = \frac{5.3\ mF\ cm^{-2}}{40\mu F.cm^{-2} per cm_{ECSA}^{2}} = 132.5\ cm_{ECSA}^{2}$$

IrO_2:

$$A_{ECSA}^{IrO2} = \frac{7.2\ mF\ cm^{-2}}{40\mu F.cm^{-2} per cm_{ECSA}^{2}} = 180.0\ cm_{ECSA}^{2}$$

Pt/C:

$$A_{ECSA}^{Pt/C} = \frac{14.0\ mF\ cm^{-2}}{40\mu F.cm^{-2} per cm_{ECSA}^{2}} = 350.0\ cm_{ECSA}^{2}$$

CoNi LDH:

$$A_{ECSA}^{CoNi\ LDH} = \frac{50.0\ mF\ cm^{-2}}{40\mu F.cm^{-2} per cm_{ECSA}^{2}} = 1250.0\ cm_{ECSA}^{2}$$

CoNiS:

$$A_{ECSA}^{CoNiS} = \frac{57.5\ mF\ cm^{-2}}{40\mu F.cm^{-2} per cm_{ECSA}^{2}} = 1437.5\ cm_{ECSA}^{2}$$

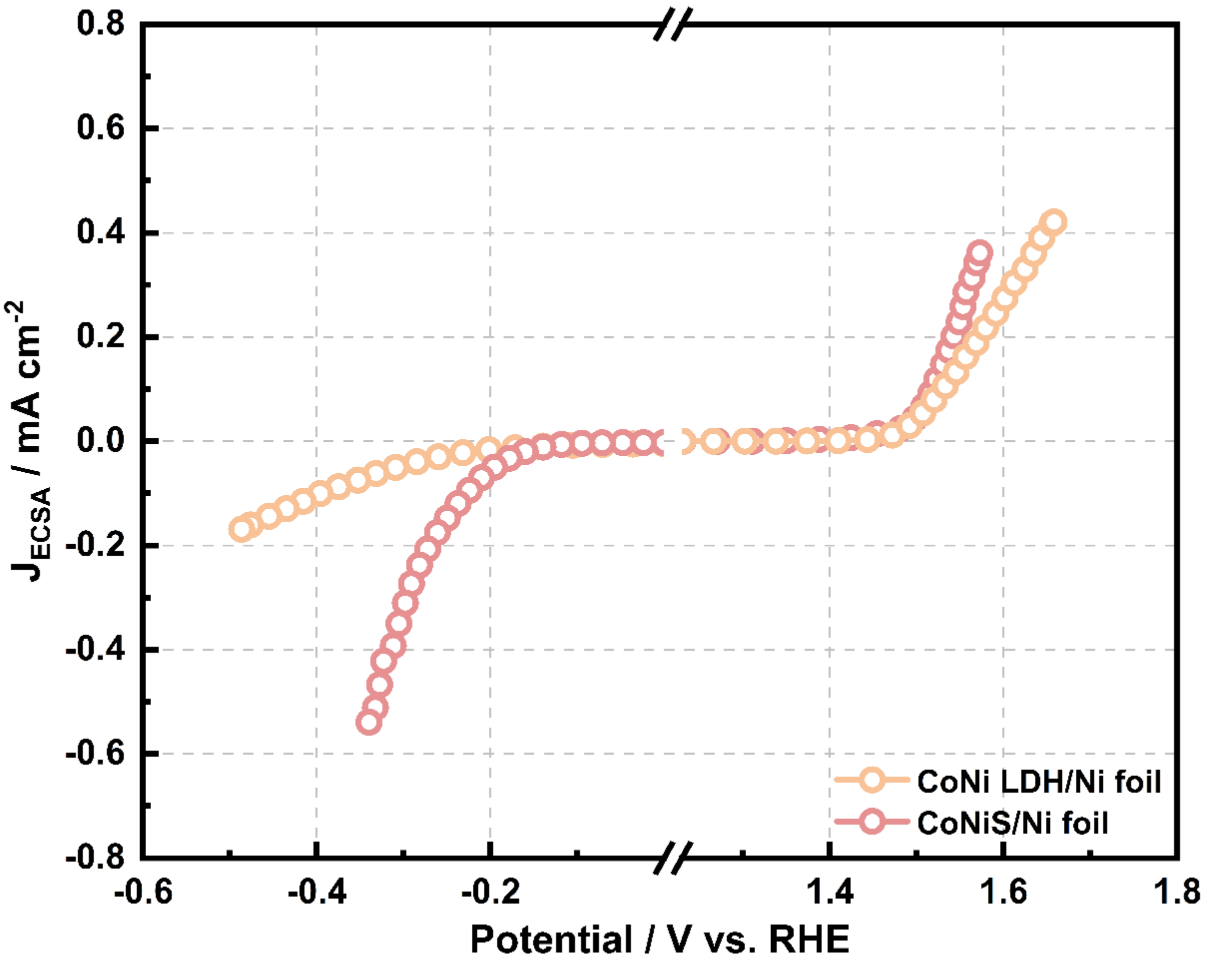

Supplementary Fig. 29 | Polarization curves normalized by ECSA. Linear sweep voltammograms (LSV) curves of CoNi LDH/Ni foil and CoNiS/Ni foil for OER and HER.

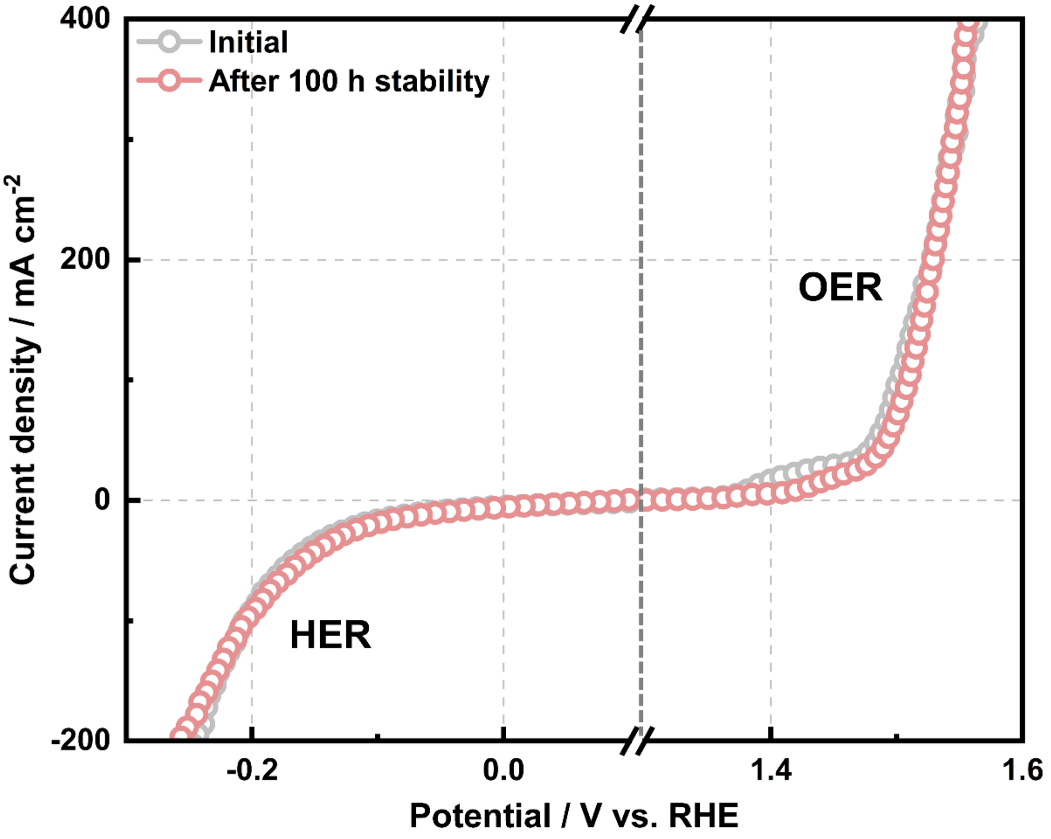

Supplementary Fig. 30 | The electrocatalytic stability evaluation. The polarization curve of CoNiS/Ni foil electrode after HER and OER stability testing, respectively.

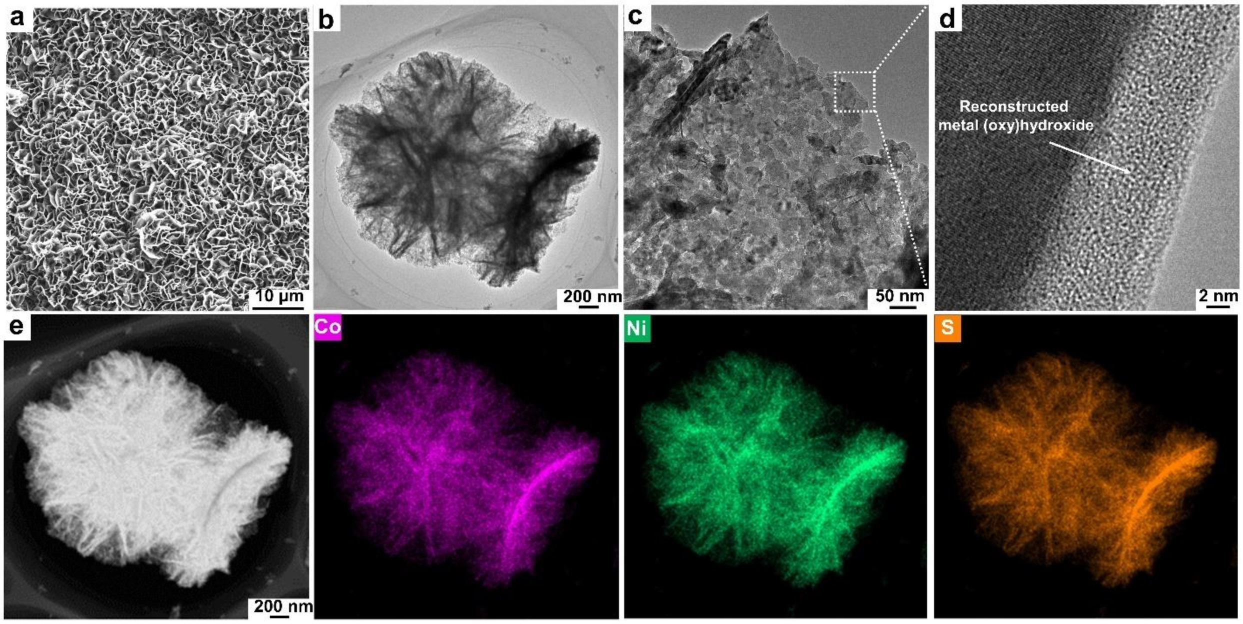

Supplementary Fig. 31 | The morphology structure characterization of CoNiS nanosheet arrays after OER stability tests. (a) FESEM image, TEM images with (b-c) low and (d) high magnifications. (e) energy-dispersive spectroscopy elemental mapping images of CoNiS nanosheet.

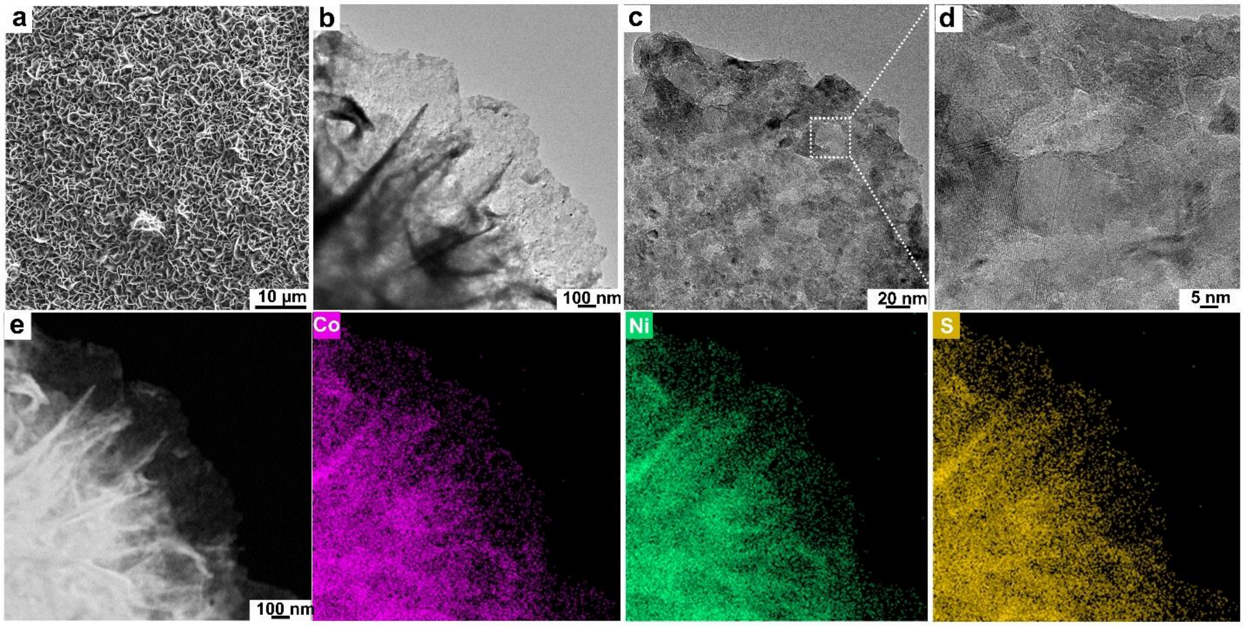

Supplementary Fig. 32 | The morphology structure characterization of CoNiS nanosheet arrays after HER stability tests. (a) SEM image, TEM images with (b-c) low and (d) high magnifications. (e) energy-dispersive spectroscopy elemental mapping images of CoNiS nanosheet.

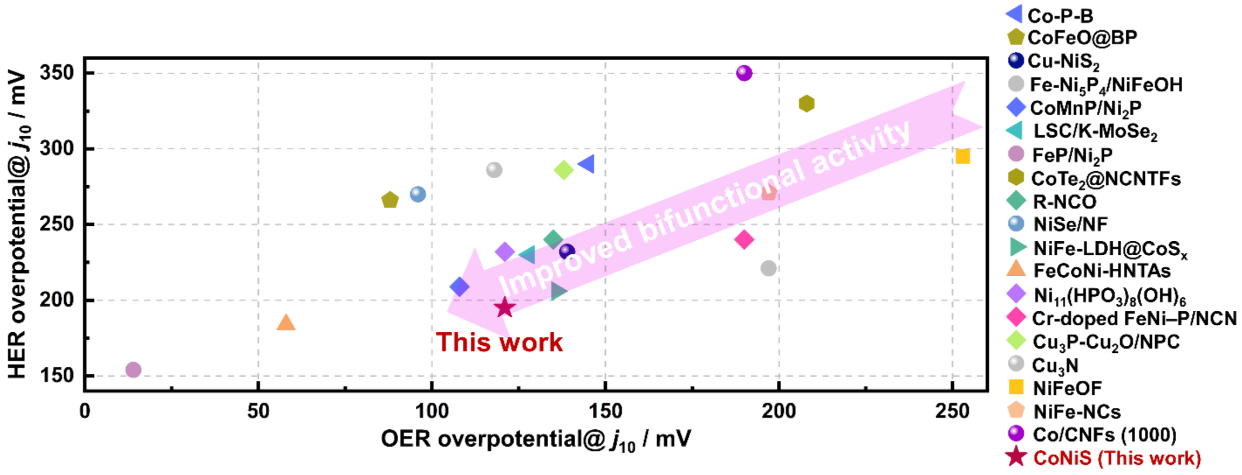

Supplementary Fig. 33 | The bifunctional activity comparison of CoNiS and reported literatures. Comparison of HER and OER overpotentials at 10 mA cm^{-2} of this work with the reported bifunctional electrocatalysts, including $Ni_{11}(HPO_3)_8(OH)_6$[1], Co-P-B[2], CoFeO@BP[3], Cr-doped FeNi-P/NCN[4], Cu-NiS_2[5], Fe-Ni_5P_4/NiFeOH[6], Cu_3P-Cu_2O/NPC[7], CoMnP/Ni_2P[8], LSC/K-$MoSe_2$[9], FeP/Ni_2P[10], Cu_3N[11], $CoTe_2$@NCNTFs[12], R-NCO[13], NiSe/NF[14], NiFeOF[15], NiFe-NCs[16], NiFe-LDH@CoS_x[17], FeCoNi-HNTAs[18] and Co/CNFs (1000)[19].

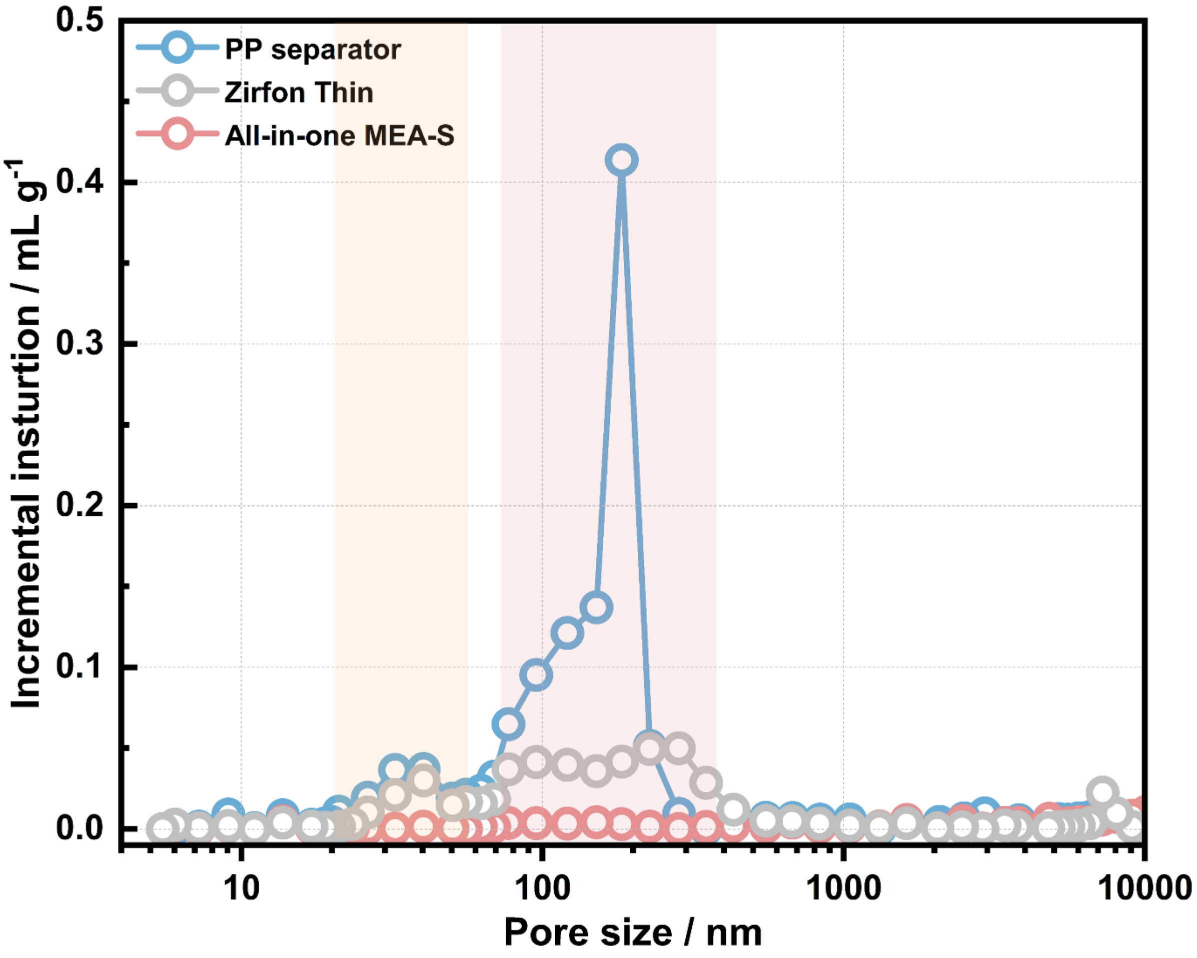

Supplementary Fig. 34 | The pore size distribution of different membranes and all-in-one MEA-S. The curves of incremental intrusion curves for pristine PP separator, Zirfon Thin and all-in-one MEA-S as a function of pore size diameter by mercury porosimeter.

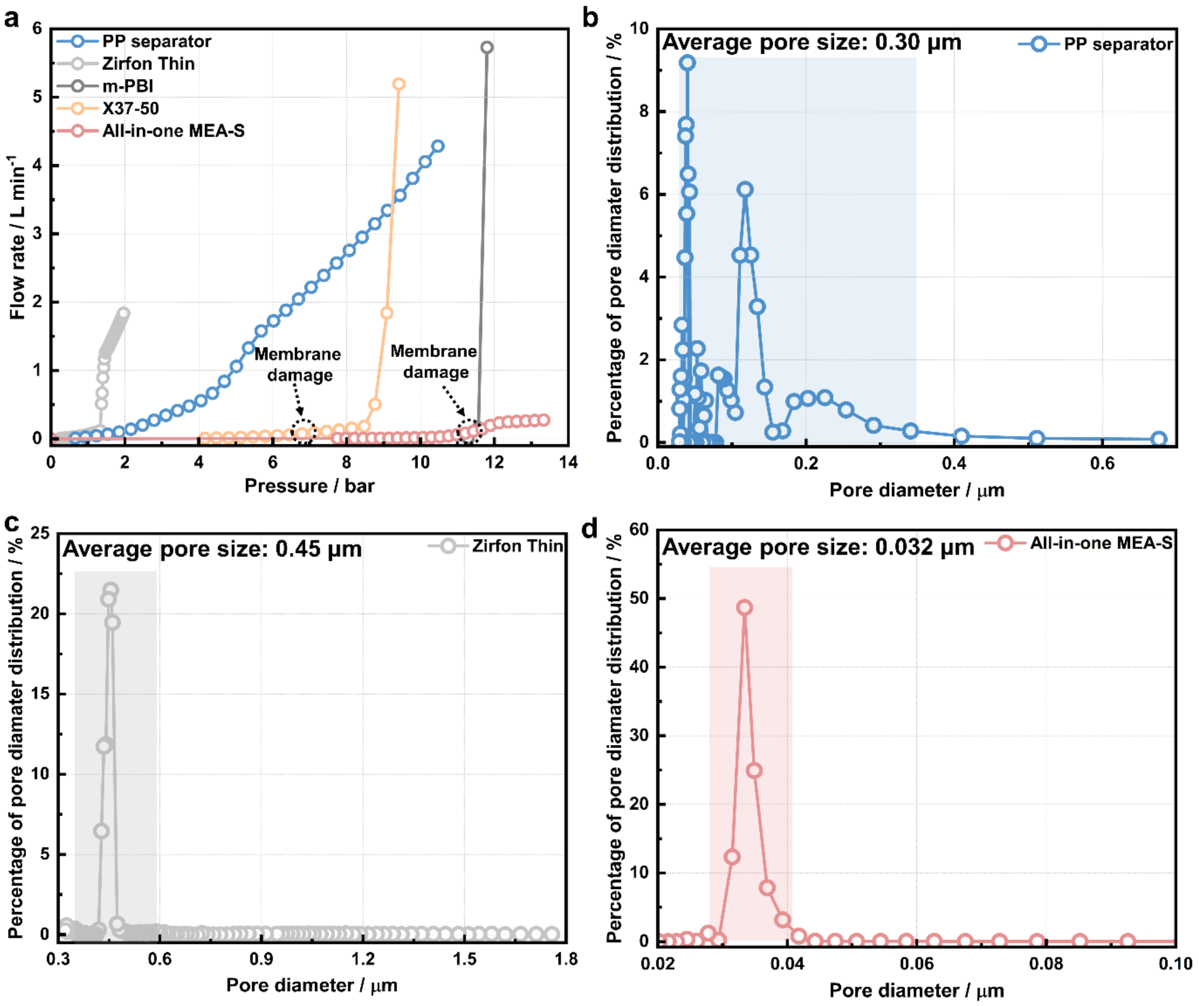

Supplementary Fig. 35 | The bubble point and pore size distribution of different membranes and all-in-one MEA-S. (a) Differential pressures and flow rates of PP separator, Zirfon Thin, m-PBI, Sustainion X37-50 and all-in-one MEA-S. (b)The pore diameter distribution of (b) PP separator, (c) Zirfon Thin and (d) all-in-one MEA-S, measured using a capillary flow porometer

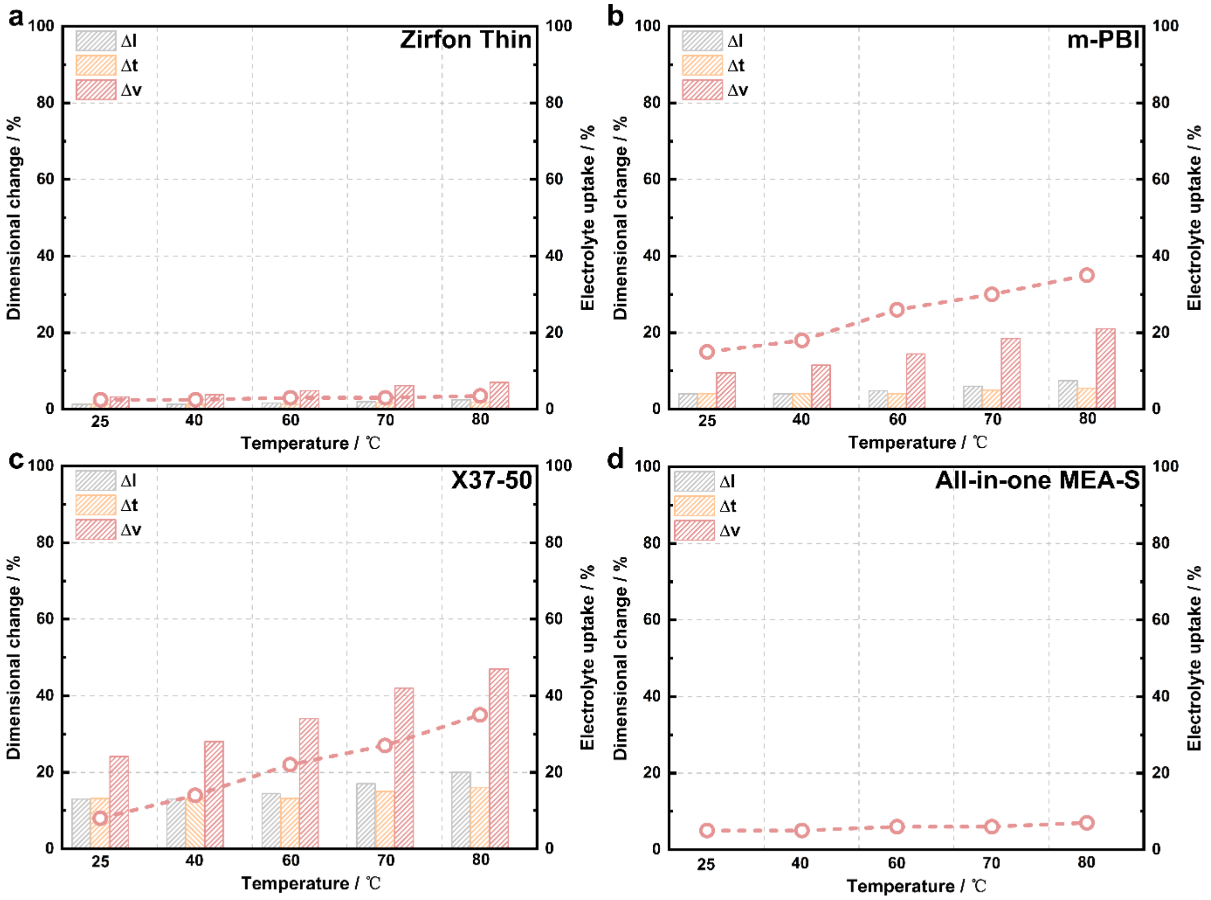

Supplementary Fig. 36 | Swelling ratio of membranes and all-in-one MEA-S. Electrolyte absorption behaviour of (a) Zirfon Thin, (b) m-PBI, (c) Sustainion X37-50I and (d) All-in-one MEA-S. The circle symbol depicts the water uptake of membranes. The dimensional variations of both membranes increase gradually with an increase in temperature.

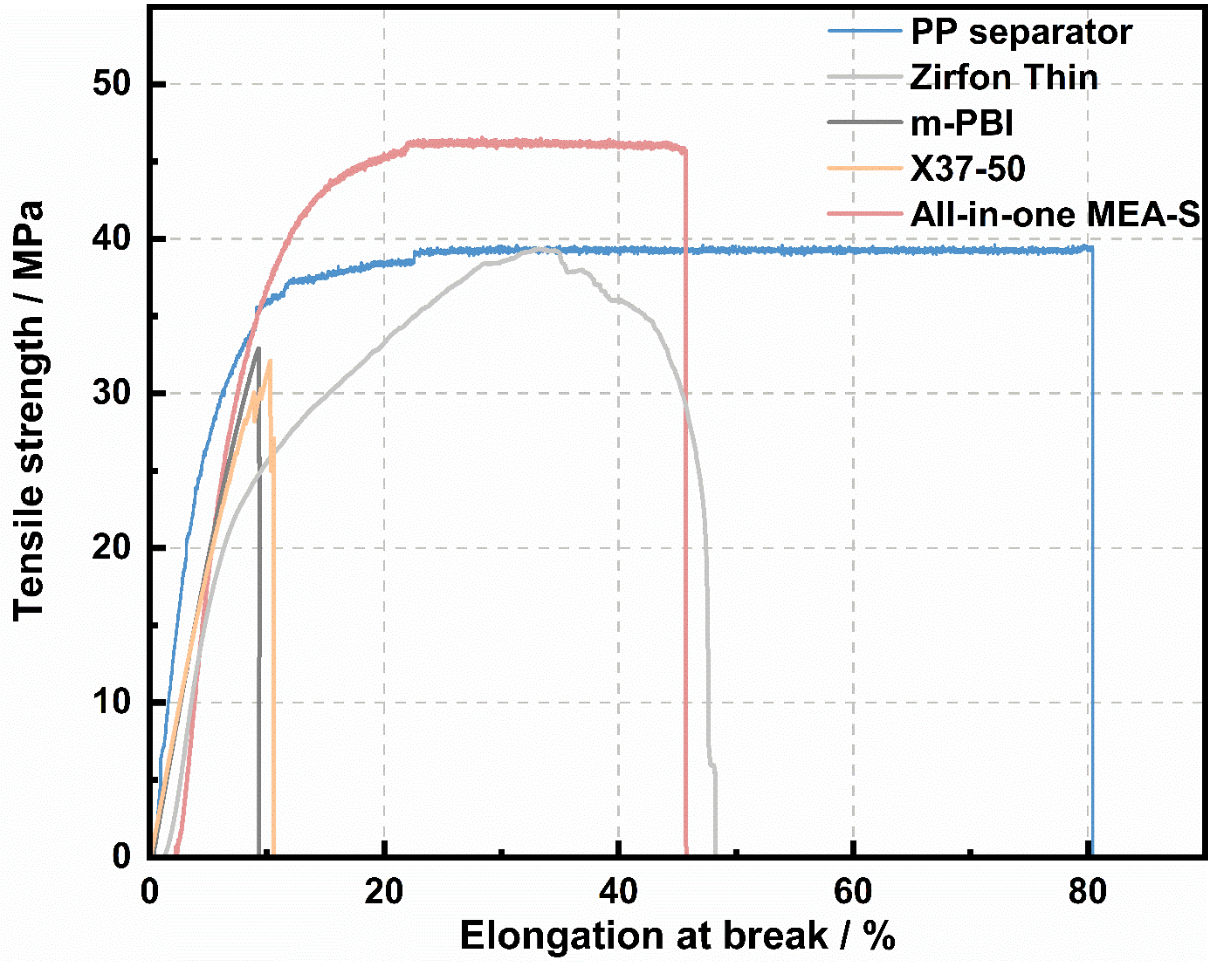

Supplementary Fig. 37 | Mechanical strength of membranes and all-in-one MEA-S. Stress-strain curves of the PP separator, commercial Zirfon Thin, m-PBI, Sustainion X37-50 membranes and all-in-one MEA-S.

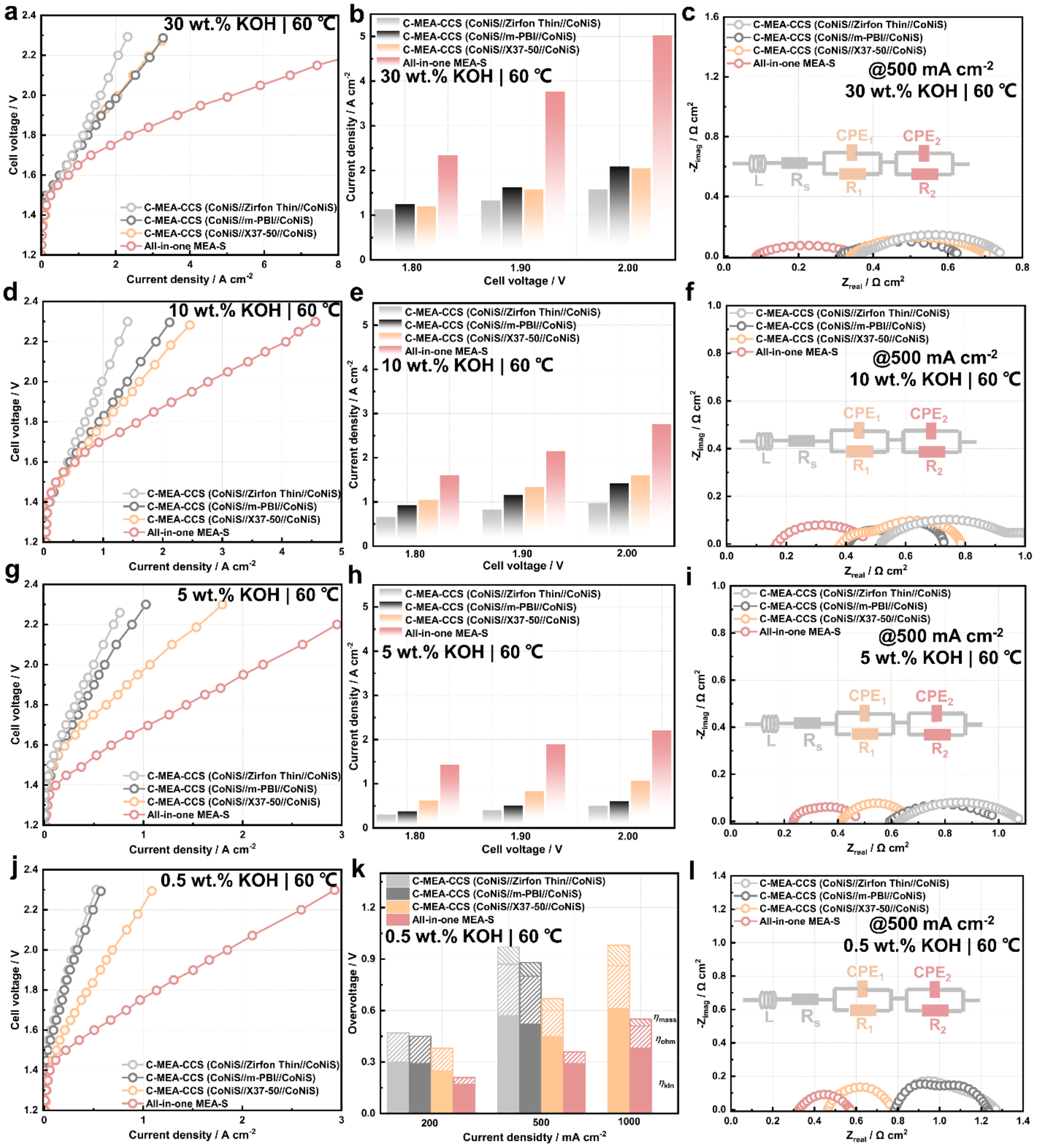

Supplementary Fig. 38 | Performance of conventional MEA-CCS (C-MEA-CCS) and all-in-one MEA-S. C-MEA-CCS (CoNiS//Zirfon Thin//CoNiS) uses Zirfon Thin and self-supported CoNiS as membrane and electrodes. C-MEA-CCS (CoNiS//m-PBI//CoNiS) uses m-PBI and self-supported CoNiS as membrane and electrodes. C-MEA-CCS (CoNiS//X37-50//CoNiS) uses Sustainion X37-50 and self-supported CoNiS as membrane and electrodes. Polarization curves of different MEAs in (a) 30 wt% KOH,

(d) 10 wt% KOH, (g) 5 wt% KOH and (j) 0.5 wt% KOH at 60 ℃. The cell voltage of different MEAs in (b) 30 wt% KOH, (e) 10 wt% KOH and (h) 5 wt% KOH at various current density. Nyquist plot of different MEAs at 500 mA cm^{-2} in (c) 30 wt% KOH, (f) 10 wt% KOH, (i) 5 wt% KOH and (i) 0.5 wt.% KOH at 60 ℃. (k) Bar diagram of overvoltage current density (200, 500 and 1000 mA cm^{-2}) of different MEAs divided by mass transfer (η_{mass}), kinetics (η_{kin}) and ohmic (η_{ohm}) in 0.5 wt.% KOH at 60 ℃.

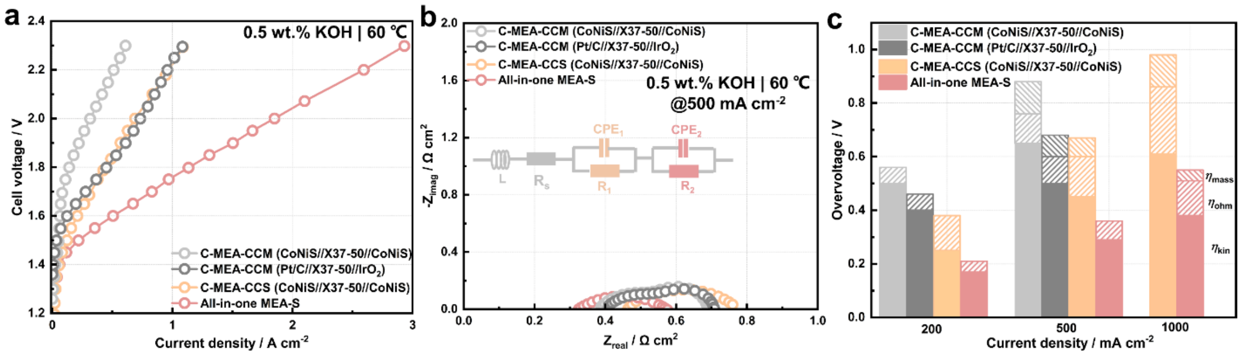

Supplementary Fig. 39 | Performance of conventional MEAs (C-MEAs) and all-in-one MEA-S. C-MEA-CCM (CoNiS//X37-50//CoNiS) uses Sustainion X37-50 and powdery CoNiS catalysts as membrane and CLs. C-MEA-CCM (Pt/C//X37-50//IrO_2) uses Sustainion X37-50, powery Pt/C and IrO_2 catalysts as membrane, cathode and anode CLs, respectively. C-MEA-CCS (CoNiS//X37-50//CoNiS) uses Sustainion X37-50 and self-supported CoNiS as membrane and electrodes. (a) Polarization curves of different MEAs in 0.5 wt.% KOH at 60 °C. (b) Nyquist plot of different MEAs at 0.5 A cm^{-2} in 0.5 wt.% KOH at 60 °C. (c) Bar diagram of overvoltage current density (200, 500 and 1000 mA cm^{-2}) of different MEAs divided by mass transfer (η_{mass}), kinetics (η_{kin}) and ohmic (η_{ohm}) in 0.5 wt.% KOH at 60 °C.

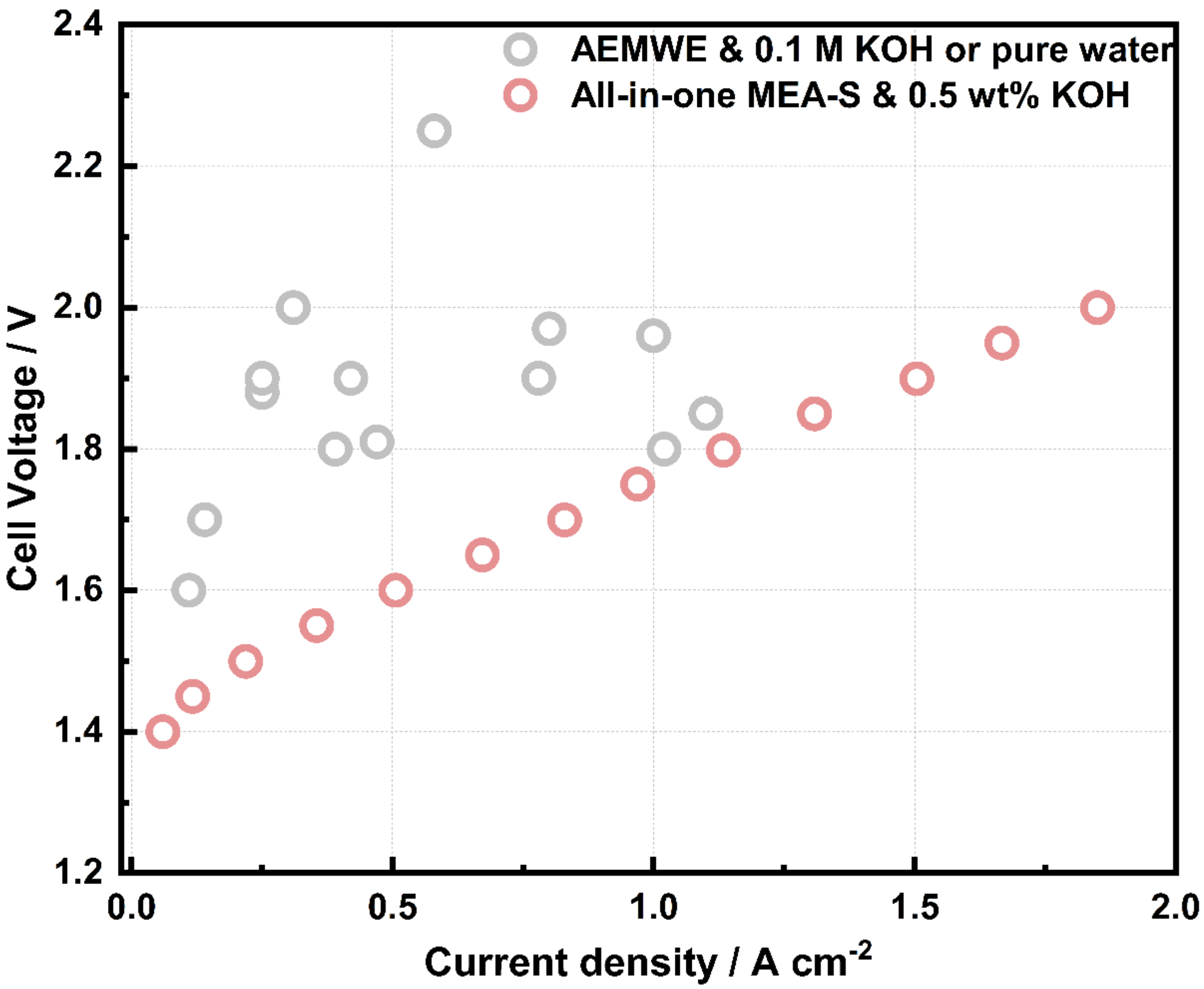

Supplementary Fig. 40 | The electrolysis performance comparison of all-in-one MEA-S and reported literatures. Comparison of cell performances of advanced alkaline water electrolysis (AWE) using all-in-one MEA-S in 0.5 wt% KOH in this study and the literatures (AEMWE fed with 0.1 M KOH and pure water).

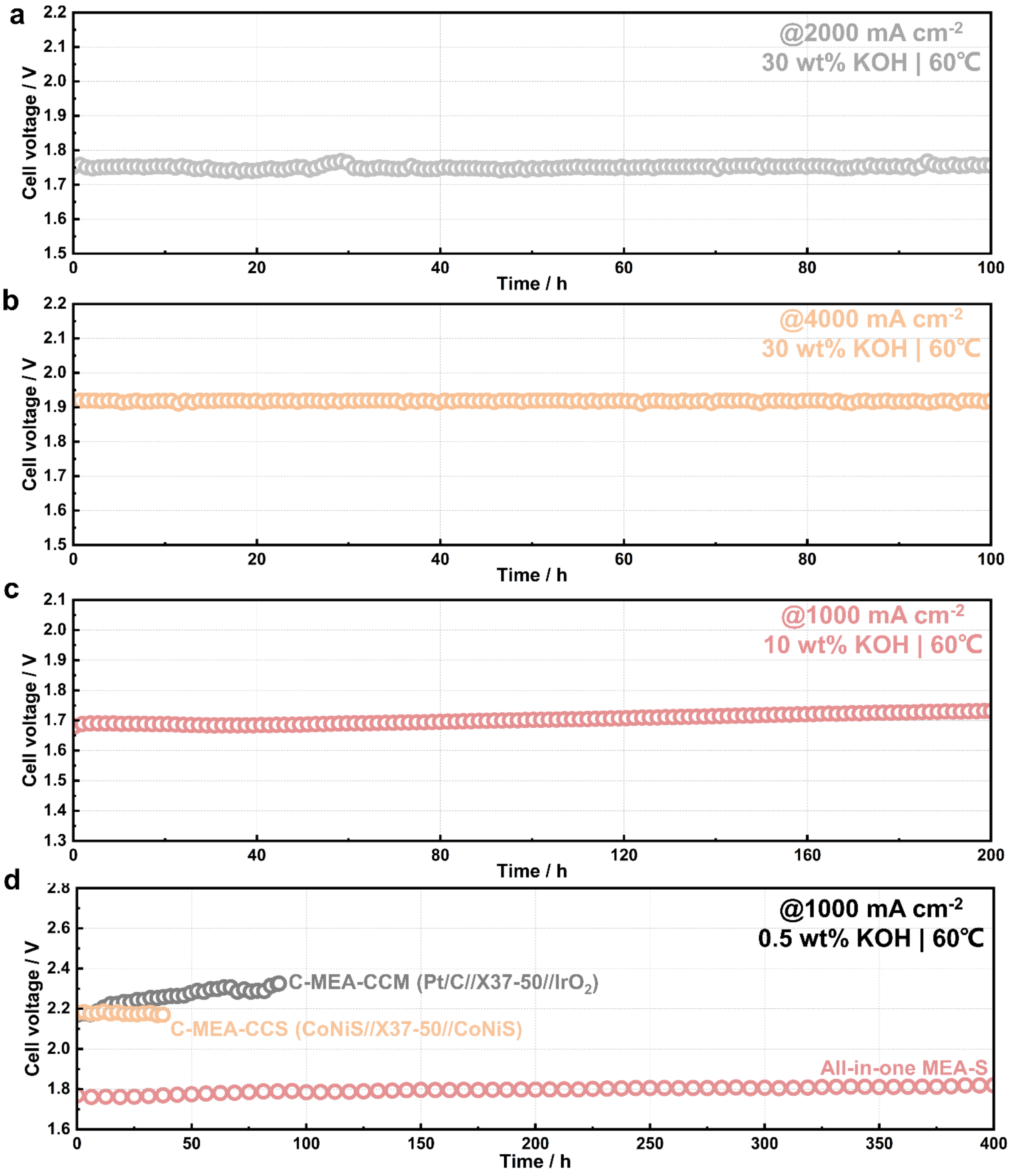

Supplementary Fig. 41 | Durability tests of the all-in-one MEA-S at different current densities and electrolyte concentration. (a) Long-term stability performance of all-in-one MEA-S at 2000 mA cm^{-2} and 60 ℃ in 30 wt% KOH electrolyte. (b) Long-term stability performance of all-in-one MEA-S at 4000 mA cm^{-2} and 60 ℃ in 30 wt% KOH electrolyte. (c) Long-term stability performance of all-in-one MEA-S at

1000 mA cm^{-2} and 60 ℃ in 10 wt% KOH electrolyte. (d) Long-term stability performance of C-MEA-CCM (Pt/C//X37-50//IrO_2), C-MEA-CCS (CoNiS//X37-50//CoNiS) and all-in-one MEA-S at 1000 mA cm^{-2} and 60 ℃ in 0.5 wt% KOH.

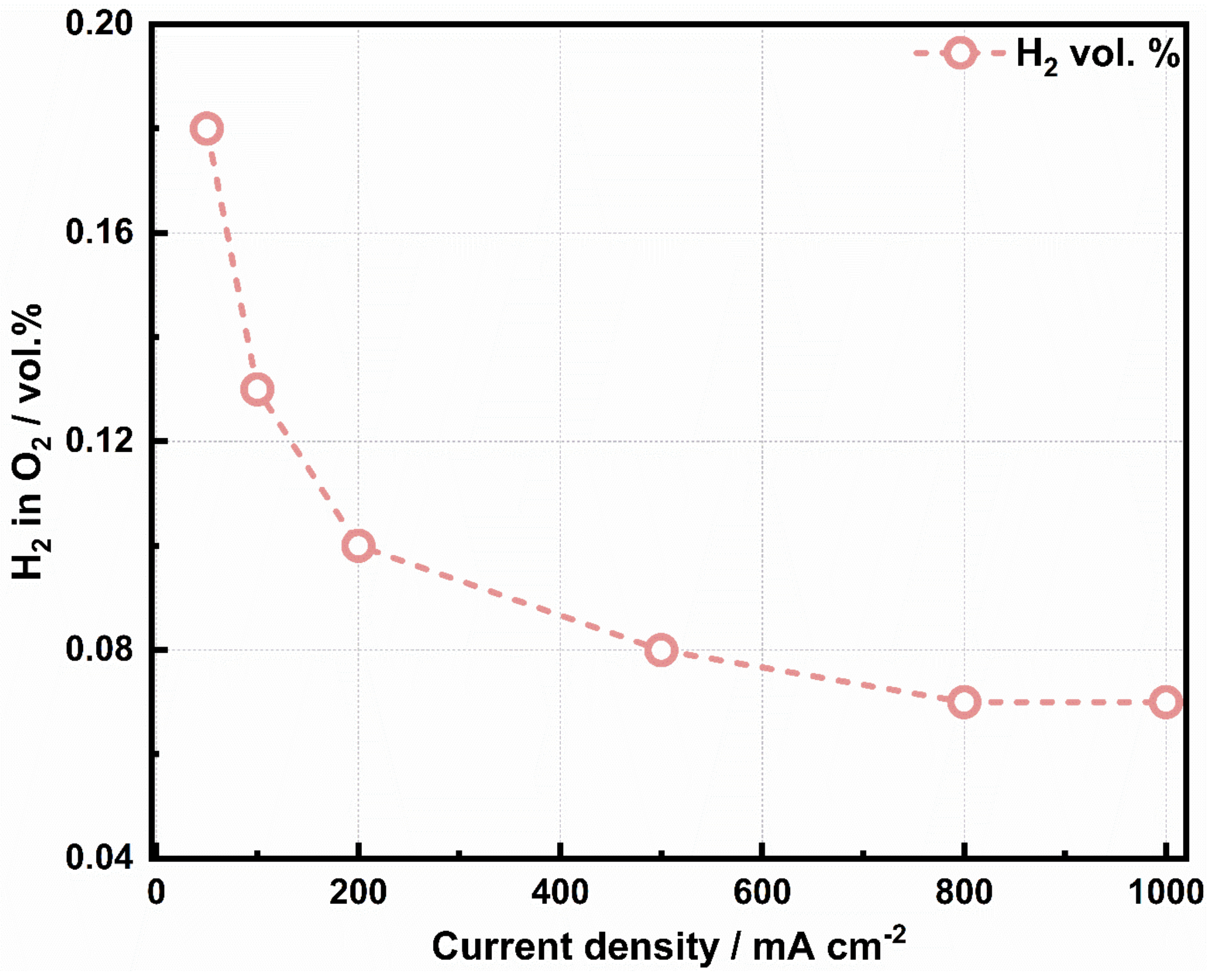

Supplementary Fig. 42 | Hydrogen crossover. The data points show the hydrogen crossover into the anodic oxygen stream of the all-in-one MEA-S as a function of current density, at 60 °C and atmospheric pressure in 30 wt% KOH. ach data point was collected after operating the cell for 30 min at the relevant current density.

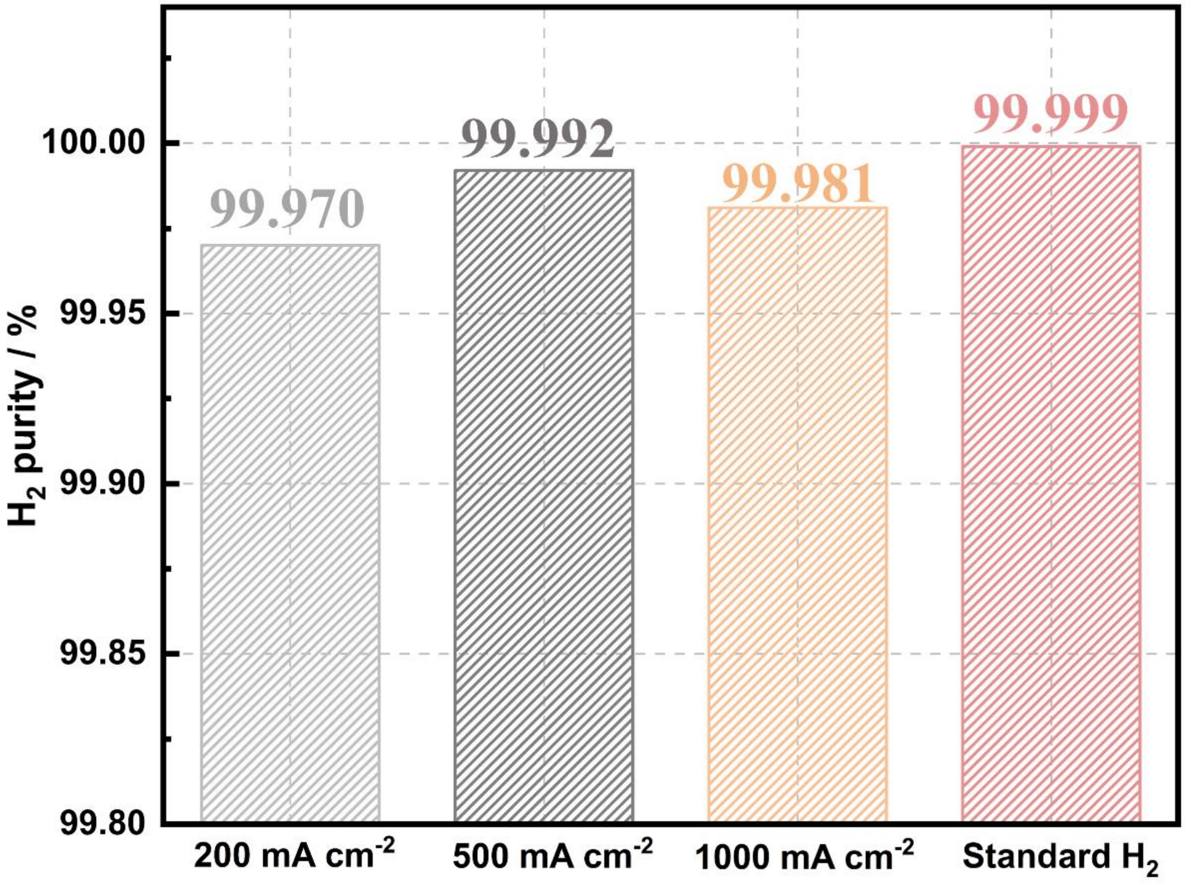

Supplementary Fig. 43 | Hydrogen gas purity generated from alkaline water electrolyzer using all-in-one MEA-S at different current densities of 200, 500 and 1000 mA cm^{-2}. Produced H_2 gas was measured by gas-chromatography (GC) and compared with standard H_2 gas (99.999 %).

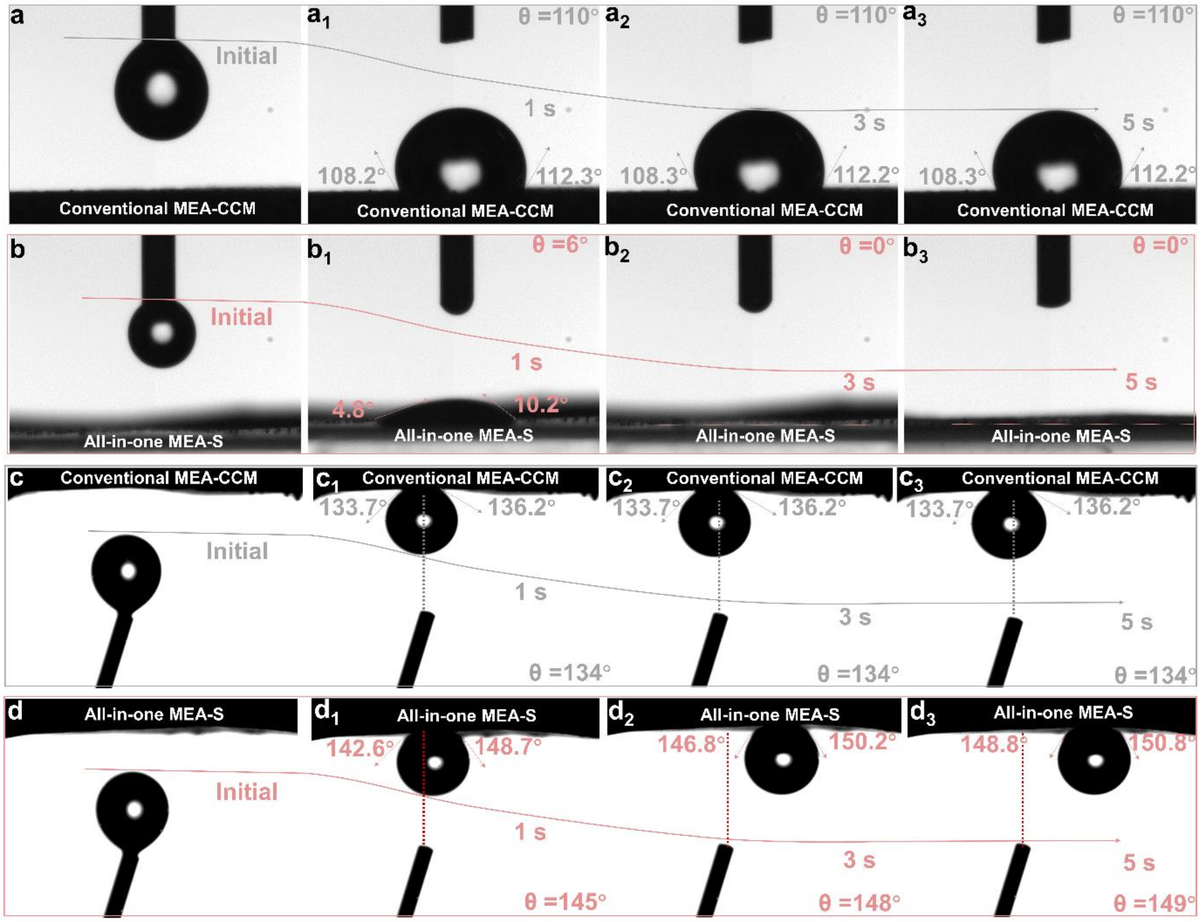

Supplementary Fig. 44 | Superhydrophilic and aerophobic measurement. The static droplets contact angles of (a) conventional MEA-CCM and (b) all-in-one MEA-S. The insets a_1-a_3 in a show the static-water-droplet measurement process where the KOH electrolyte droplets could be captured on the conventional MEA-CCM surface with passage of time. The air-bubble contact angles of (c) conventional MEA-CCM and (d) all-in-one MEA-S. The inset c_1-c_3 in a show the air-bubble measurement process where the air-bubble could be capture on the conventional MEA-CCM surface with the passage of time.

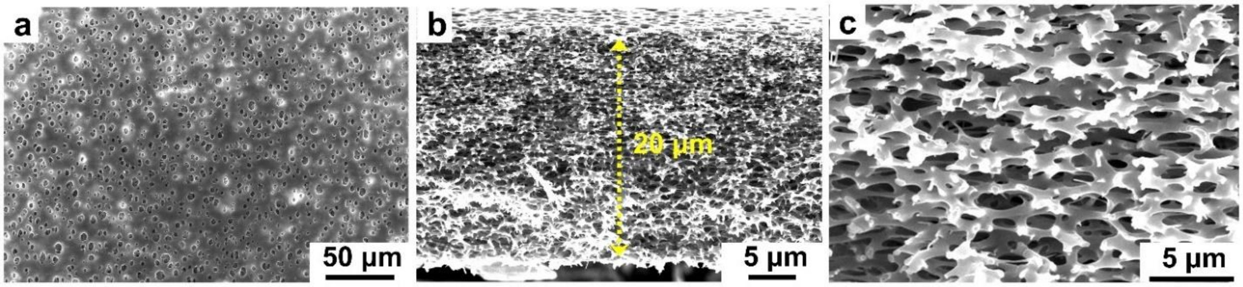

Supplementary Fig. 45 | Morphological structure characterization of PES porous membrane by SEM. (a) The surface morphology of PES porous membrane. (b-c) The cross section morphology of PES porous membrane.

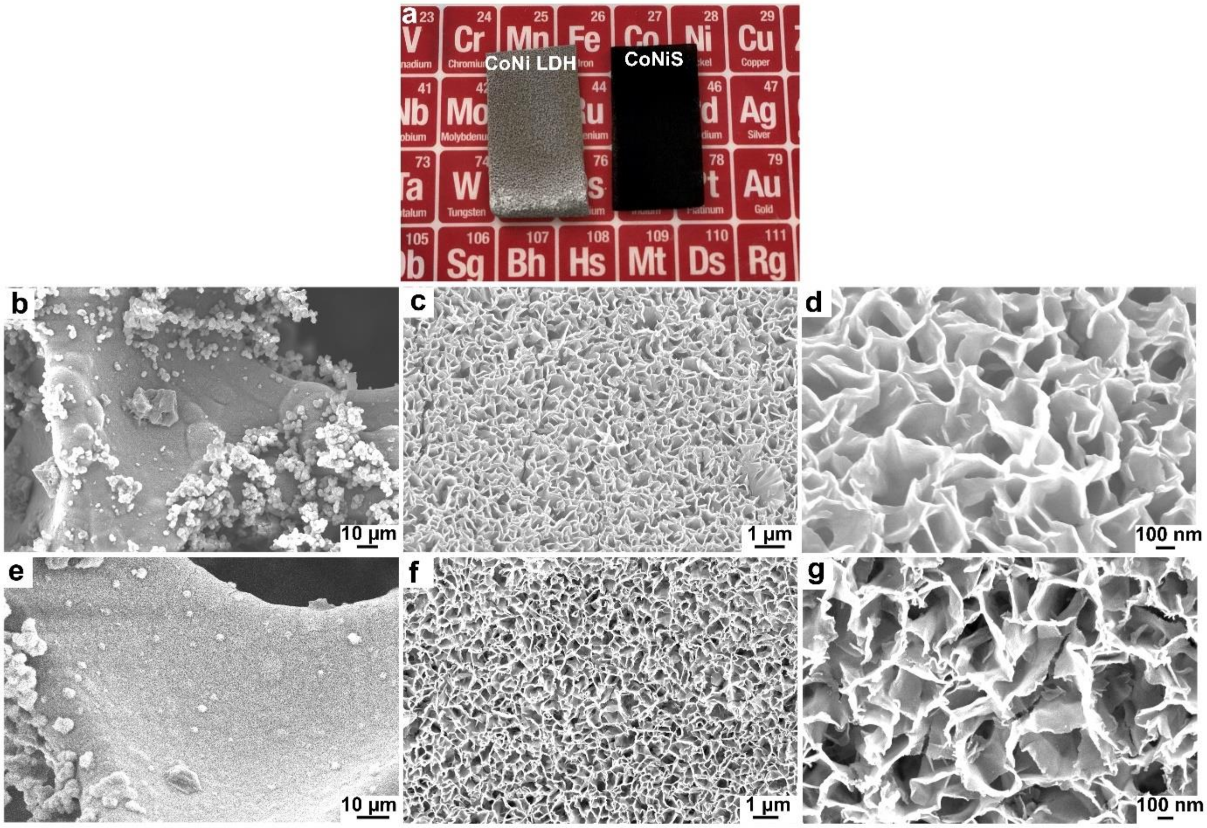

Supplementary Fig. 46 | Morphological structure characterization of CoNi LDH/Ni foam and CoNiS/Ni foam by SEM. (a) The optical image of CoNi LDH/Ni foam and CoNiS/Ni foam. The SEM images of CoNi LDH/Ni foam with (b-c) low and (d) high magnifications. The SEM images of CoNiS/Ni foam with (e-f) low and (g) high magnifications.

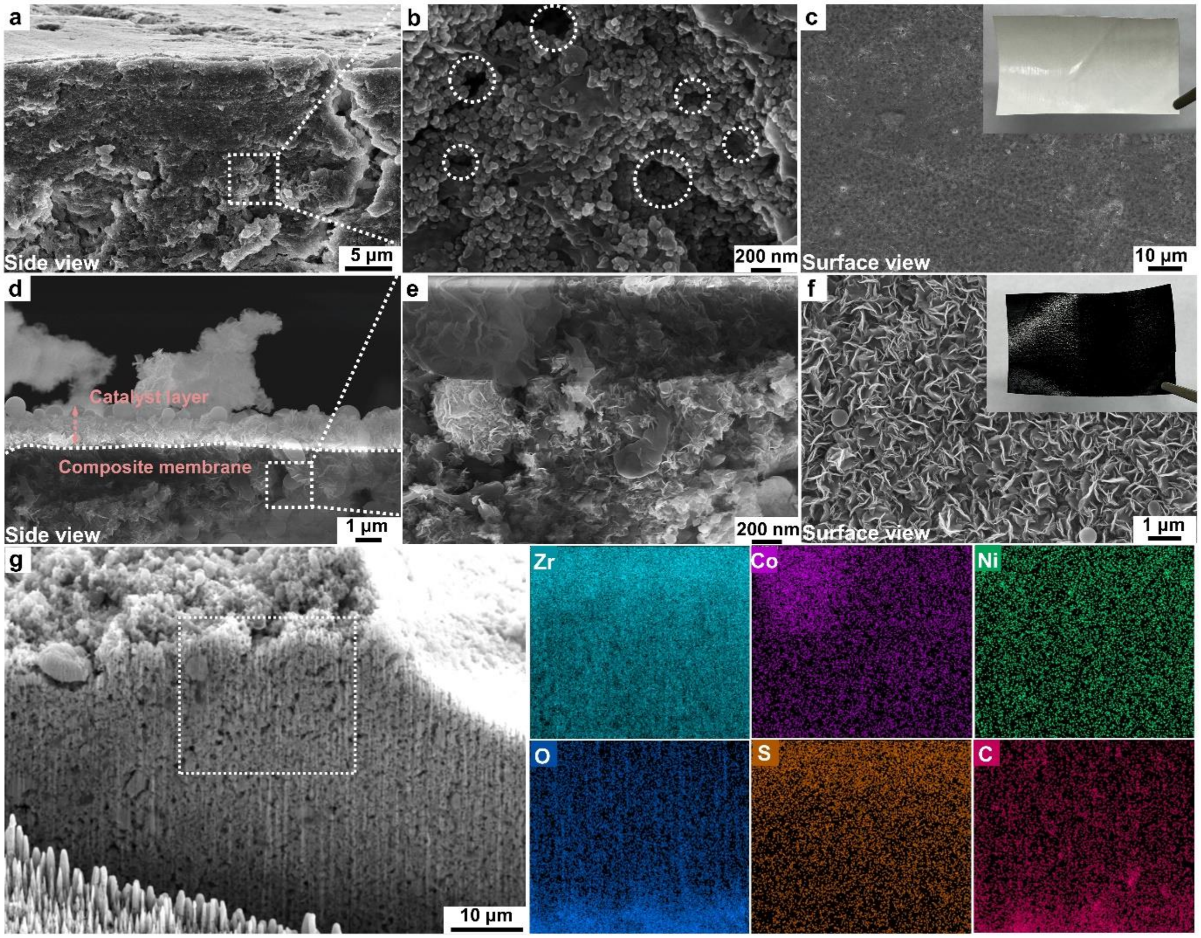

Supplementary Fig. 47 | Morphological structure characterization of all-in-one MEA prepared using commercial Zirfon Thin membrane. (a-b) Cross-sectional and (c) surface SEM image of porous Zirfon membrane (inset shows the optical image). (d-e) Cross-sectional and (f) surface SEM image of all-in-one MEA-S based on Zirfon membrane (inset shows the optical image). (g) Cross-sectional SEM image of all-in-one MEA-S based on Zirfon membrane prepared by focused ion beam (FIB) and corresponding EDX mapping.

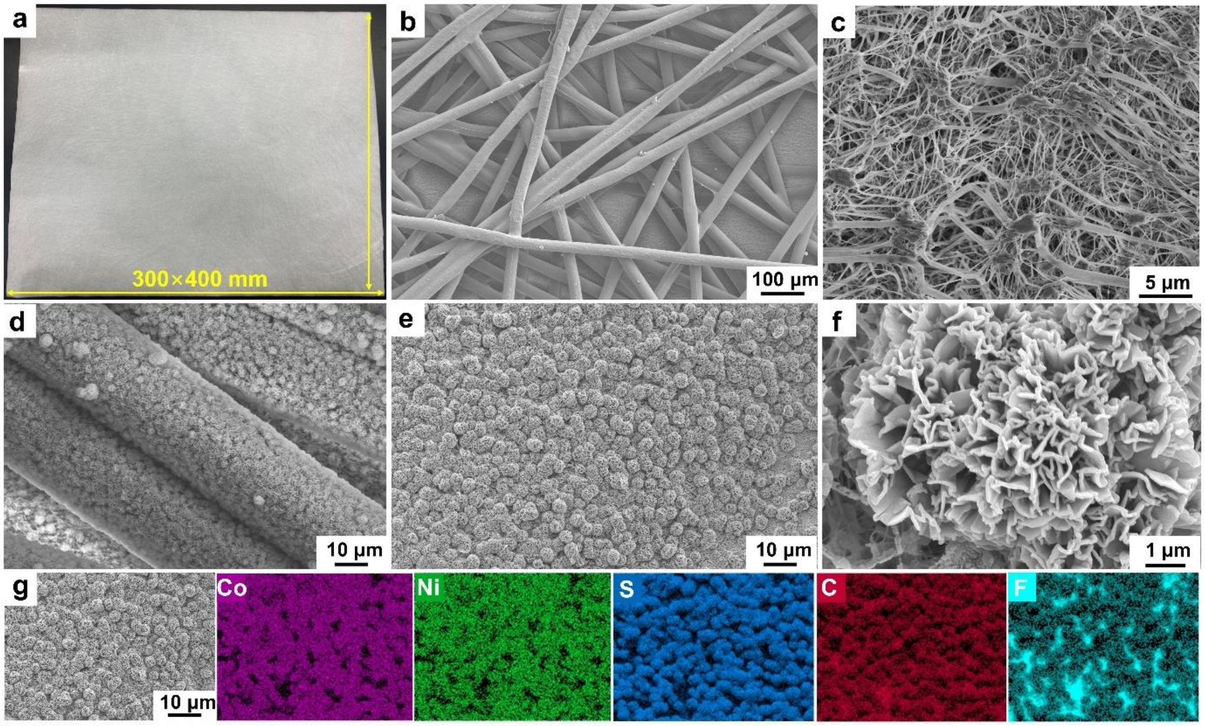

Supplementary Fig. 48 | Morphological structure characterization of all-in-one MEA-R. (a) The optical image of reinforced PTFE porous membranes. The surface morphologies of (b) reinforced PP side and (c) PTFE side. The catalyst layer morphologies of all-in-one MEA-R: (d) reinforced PP side and (e-f) PTFE side. (g) EDX mapping images of catalyst layer in all-in-one MEA-R

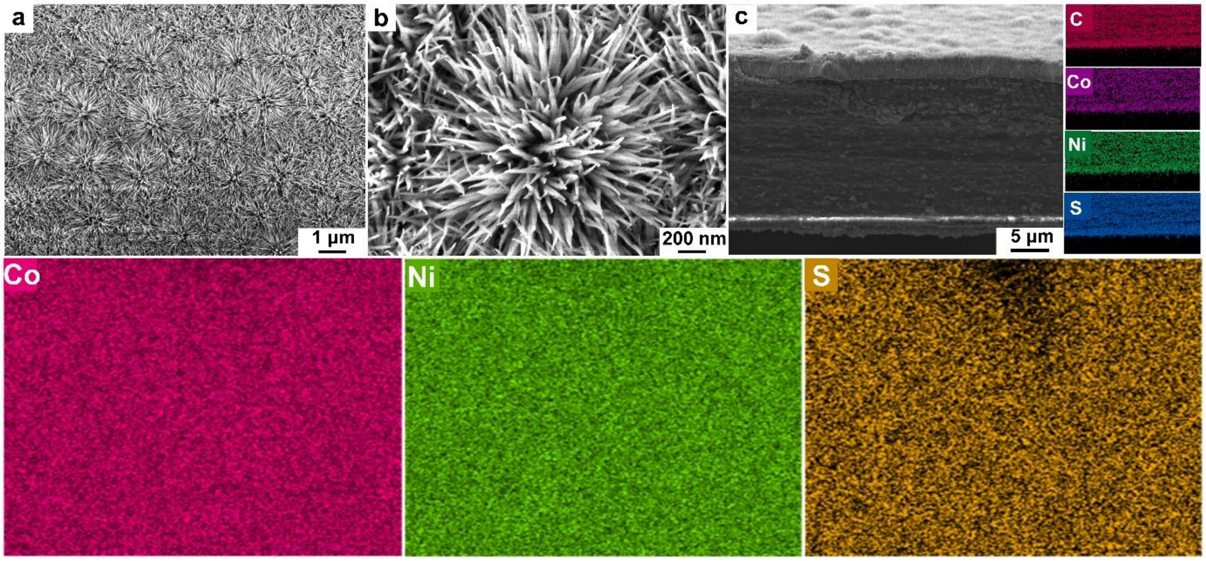

Supplementary Fig. 49 | Morphological structure characterization of all-in-one MEA-N. (a-b) The CL surface morphologies of all-in-one MEA (CoNiS NW). (c) The cross section morphologies of all-in-one MEA (CoNiS NW) and corresponding EDX mapping.

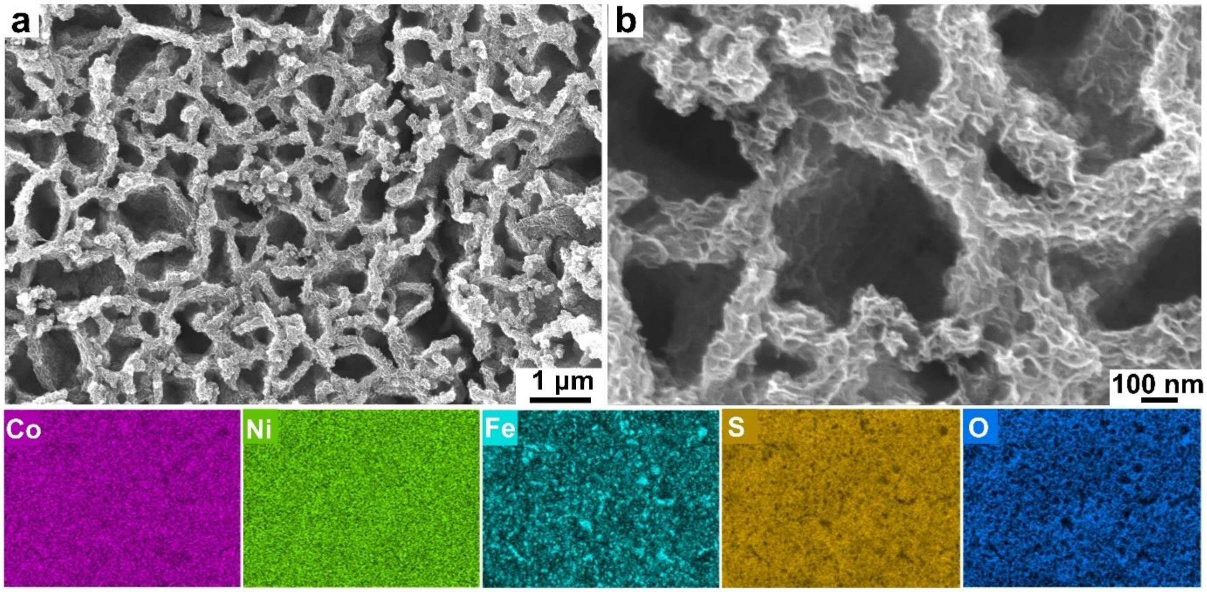

Supplementary Fig. 50 | Morphological structure characterization of all-in-one MEA (CoNiS@FeNi LDH). (a-b) The catalyst layer morphologies of all-in-one MEA based on CoNiS@FeNi LDH, and corresponding EDX mapping.

Supplementary Table 1 | Summary for the porosity and pore size of different membranes and all-in-one MEA-S.

	Porosity / %	BET pore size /μm	MP pore size /μm	CFP pore size /μm
Porous PP	42	0.34	0.36	0.30
Zirfon Thin	52	0.42	0.40	0.45
All-in-one MEA-S	4	0.04	0.05	0.03

Supplementary Table 2 | Advantages and disadvantages of alkaline water electrolysis using conventional MEAs and all-in-one MEA

ALWE using MEA-CCS	AEMWE using MEA-CCS	AEMWE using MEA-CCM	Alkaline water electrolysis using all-in-one MEA
Commercial Zirfon Thin	Sustainion X37-50	Sustainion X37-50	PP/CoNiS composite membrane
Self-supported electrodes	Self-supported electrodes	Powdery CLs	3D-ordered CLs
High chemical stability of separator	Membrane degradation	Membrane dagradation	High chemical stability of composite membrane
OH^- transport by pore of separator	OH^- transport by tethered cationic groups	OH^- transport by tethered cationic groups	OH^- transport by pore of composite membrane
High gas permeation	Low gas permeation	Low gas permeation	Low gas permeation
Corrosive liquid electrolyte (30 wt.% KOH)	Pure water or low concentrated electrolyte (1 M KOH)	Pure water or low concentrated electrolyte (1 M KOH)	Low concentrated electrolytes (0.5~30 wt% KOH)
High interfacial resistance, especially at low concentrated KOH	High interfacial resistance, especially at low concentrated KOH	Low interfacial resistance	Low interfacial resistance
3D-ordered and porous CLs	3D-ordered and porous CLs	Inordered and dense CLs	3D-ordered and porous CLs

Supplementary Table 3 | Comparison of cell performances of advanced alkaline water electrolysis (AWE) using all-in-one MEA-S in 5 wt% and 30 wt% KOH in this study and the literatures (ALWE, AEMWE using precious platinum group metal (PGM) free catalysts and PEMWE).

	Anode	Cathode	Membrane	Electrolyte	Current density / A cm^{-2}	Cell voltage / V	Ref.
ALWE	IrO_2	Pt/C	NPBI	6 M KOH	1.50	1.92	20
	NiFe LDH	Raney Ni	Zirfon-type	30 wt% KOH	0.60	1.70	21
	Ni foam	Ni foam	PVA/ABPBI	15 wt% KOH	0.50	1.93	22
	NiFe LDH	Raney Ni	Zirfon-type	30 wt% KOH	1.40	2.10	23
	NiFe LDH	Raney Ni	cPVA/Zirfon	30 wt% KOH	1.00	2.05	24
	Ni	Ni	Porous PFSA	30 wt% KOH	0.20	2.12	25
	Ni/MoN/rNS	Ni/MoN/rNS	PPS	30 wt% KOH	0.40	1.85	26
AEMWE using PGM free catalysts	$Ni_2P@FePO_xH_y$	$MoNi_4/MoO_2$	AEM	1 M KOH	0.50	1.75	27
	NiFe	Ni	FAA-3-50	1 M KOH	0.40	2.00	28
	Ni felt	Ni felt	Aemion™	1 M KOH	0.30	2.10	29
	NiFeCo LDH	NiFeCoP	X37-50 Grade T	1 M KOH	0.50	1.72	30
	$NiFe_2O_4$	NiFeCO	TPNPiQA	1 M KOH	0.85	2.20	31
	NiFe	NiFe	PFTP-13	1 M KOH	0.92	1.80	32
	NiFe LDH	NiMo	PVBC-Mpy/PEK	1 M KOH	0.90	2.13	33
	$NiCo_2O_4$	$NiCo_2O_4$	PAni	1 M KOH	0.37	2.00	34
PEMWE	Ir	Pt/C	Nafion 115	Pure water	1.95	1.95	35
	IrO_2	Pt/C	Nafion 117	Pure water	1.50	1.80	36
	IrO_2	Pt/C	Multiblock PEM	Pure water	3.00	1.87	37
	IrO_2	Pt/C	Nafion 117	Pure water	2.00	1.80	38
	IrO_2	CuNiMo	Nafion 212	Pure water	1.45	1.90	39
	IrO_2	Pt/C	Nafion 115	Pure water	3.80	2.20	40

Supplementary Table 4 | Comparison of cell performances of advanced alkaline water electrolysis (AWE) using all-in-one MEA-S in 0.5 wt% KOH in this study and the literatures (AEMWE fed with 0.1 M KOH and pure water).

	Anode	Cathode	Membrane	Electrolyte	Current density / A cm^{-2}	Cell voltage / V	Ref.
AEMWE	a-NiFeOOH	$MoNi_4/MoO_2$	FAA-3-50	Pure water	0.25	1.88	41
	NiFe	Ni	FAA-3-50	0.1 M KOH	0.31	2.00	28
	Ni-LCO/C	$Cu_{0.5}Co_{2.5}O_4$	X37-50 Grade T	0.1 M KOH	0.78	1.90	42
	NiFe LDH	Pt/C	HWU-AEM	Pure water	0.25	1.90	43
	$NiCoFeO_x$	Pt black	AEM	Pure water	0.58	2.25	44
	NiFe-BTC-GNPs	$MoNi_4/MoO_2$	FAA-3-PK-130	0.1 M KOH	1.10	1.85	45
	NiCoFe-NDA	Pt/C	X37-50 Grade T	0.1 M KOH	0.42	1.90	46
	IrO_2	Pt/C	PiperION	Pure water	0.80	1.97	47
	IrO_2	Pt/C	QPC-TMA	Pure water	0.14	1.70	48
	FeNiOOH-F	Pt/C	PAP-TP-85	Pure water	1.02	1.80	49
	$Ir-Ni/Mo_5N_6$	$Ir-Ni/Mo_5N_6$	X37-50 Grade T	Pure water	0.11	1.6	50
	Acta 3030	Acta 4030	A201	1 wt% K_2CO_3	0.47	1.81	51
	IrO_2	Pt black	A201	Pure water	0.39	1.80	52
	IrO_2	Pt/C	PFOTFPh	Pure water	1.00	1.96	53

Supplementary Table 5 | Comparison of operating parameters of stacks of 200 of the individual cells at 60 ℃

Cell stack type	Fixed current density / A cm^{-2}	Number of cells	Current / A	Cell stack voltage / V	Total power / kW	Electrolyte / KOH wt%
Conventional MEA-CCM (Pt/C//X37-50//IrO_2)	1.0	200	200	392	78.4	5
Conventional MEA-CCS (CoNiS//Zirfon Thin//CoNiS)	1.0	200	200	356	71.2	30
All-in-one MEA-S	1.0	200	200	330	66.0	5
All-in-one MEA (CoNiS@FeNi LDH)	1.0	200	200	314	62.8	30

Supplementary Table 6 | Comparison of reported hydrogen crossover with conventional separators.

Separator	Electrolyte	Temperature / °C	Pressure / bar	Current density / A cm^{-2}	Concentration (H_2 in O_2) / vol.%	Reference
All-in-one MEA-S	30 wt% KOH	60	1 atm	0.05	0.18	This work
All-in-one MEA-S	30 wt% KOH	60	1 atm	0.20	0.10	This work
Zirfon PERL UTP 500	30 wt% KOH	80	10	0.40	0.21	[54]
PTFE/LDH	1 M KOH	80	1 atm	0.50	1.30	[55]
Zirfon	30 wt% KOH	80	1 atm	0.10	2.42	[24]
cPVAZ-30	30 wt% KOH	80	1 atm	0.10	0.32	[24]
cPVAZ-30	30 wt% KOH	80	1 atm	0.20	0.27	[24]
Zirfon	30 wt% KOH	80	0.3	0.20	1.59	[21]
Z80_300um	30 wt% KOH	80	0.3	0.05	1.24	[21]
Z80_300um	30 wt% KOH	80	0.3	0.20	0.26	[21]
Z80C5	10 wt% KOH	80	0.1	0.05	0.26	[56]
Z80C5	10 wt% KOH	80	0.1	0.20	0.21	[56]
Z80C5	10 wt% KOH	80	0.3	0.05	0.75	[56]
Nafion 117	DI water	80	1 atm	0.40	1.91	[57]
Nafion 212	DI water	80	1 atm	1.40	1.91	[57]

Supplementary Table 7 | The comparison of energy efficiency at the current density of 1000 mA cm^{-2} for all-in-one MEAs and recently reported alkaline water electrolyzers

Anode	Cathode	membrane	Voltage@1000 mA cm^{-2}	Energy efficiency / %	Reference
NiFe	Ni	FAA-3-50	2.3	63.91	[28]
FeNiPS	FeNiPS	FAA-3-50	1.75	84.00	[58]
NiFeO	Pt/C	X37-50 Grade T	1.76	83.52	[9]
NiAlMo	NiAlMo	HMT-PBI	1.82	80.77	[59]
IrO_2	Pt/C	PFOTFPh-TMA	1.78	82.58	[53]
$NiMoO_2$	NiCoFe	Fumapem-3-PE-30	1.96	75.00	[60]
IrO_2	Pt/C	PiperION	1.68	87.50	[47]
IrO_2	PtRu/C	HTMA-DAPP	1.66	88.55	[61]
NiFe LDH	Pt/C	X37-50	1.7	86.47	[62]
Co	PtNi	AEM	1.9	77.37	[63]
NiCoFeO	Pt	AEM	2.5	58.80	[44]
NiFe LDH	Pt/C	X37-50 Grade T	1.58	93.04	[64]
NiFeOOH	Pt/C	FAA-3-50	1.62	90.74	[65]
$CoSb_2O_6$	Pt/C	FAA-3-50	2	73.50	[66]
$CuCo_2O_4$	Pt/C	X37-50 Grade T	1.83	80.32	[67]
Fe-NiMo	NiMo	X37-50 Grade T	1.57	93.63	[68]
FeNi LDH	CoP	PTFE/LDH	1.8	81.67	[55]
IrO_2	Ru_2P	X37-50	1.87	78.61	[69]
FeNi LDH	Raney Ni	Cellulose–blended Zirfon	1.72	85.47	[23]
CoFe	Pt/C	FAA-3-50	1.73	84.97	[70]
IrO_2	Pt/C	FAA-3-50	1.78	82.58	[71]
CuCoO	Co_3S_4	X37-50	2.2	66.82	[72]
IrO_2	CuCoP	X37-50	2.03	72.41	[73]
FeNi LDH	Pt/C	HWU-AEM	1.92	76.56	[43]
Ni	Ni	PVBC-Mpy/PEK-cardo	2.2	66.82	[33]
CoNiS@FeNi LDH	CoNiS@FeNi LDH	All-in-one MEA	1.57	94.00%	This work

Supplementary References

1. Menezes PW, *et al.* A structurally versatile nickel phosphite acting as a robust bifunctional electrocatalyst for overall water splitting. *Energy & Environmental Science* **11**, 1287-1298 (2018).

2. Chunduri A, *et al.* A unique amorphous cobalt-phosphide-boride bifunctional electrocatalyst for enhanced alkaline water-splitting. *Applied Catalysis B: Environmental* **259**, 118051 (2019).

3. Li X, *et al.* Adaptive Bifunctional Electrocatalyst of Amorphous CoFe Oxide @ 2D Black Phosphorus for Overall Water Splitting. *Angew Chem Int Ed Engl* **59**, 21106-21113 (2020).

4. Wu Y, *et al.* Cr-Doped FeNi-P Nanoparticles Encapsulated into N-Doped Carbon Nanotube as a Robust Bifunctional Catalyst for Efficient Overall Water Splitting. *Adv Mater* **31**, e1900178 (2019).

5. Dinh KN, *et al.* Electronic Modulation of Nickel Disulfide toward Efficient Water Electrolysis. *Small* **16**, e1905885 (2020).

6. Li C-F, Zhao J-W, Xie L-J, Wu J-Q, Li G-R. Fe doping and oxygen vacancy modulated Fe-Ni5P4/NiFeOH nanosheets as bifunctional electrocatalysts for efficient overall water splitting. *Applied Catalysis B: Environmental* **291**, 119987 (2021).

7. Zhu J, *et al.* Gram-Scale production of Cu3P-Cu2O Janus nanoparticles into nitrogen and phosphorous doped porous carbon framework as bifunctional electrocatalysts for overall water splitting. *Chemical Engineering Journal* **427**, 130946 (2022).

8. Liu M, *et al.* Hierarchical superhydrophilic/superaerophobic CoMnP/Ni2P nanosheet-based microplate arrays for enhanced overall water splitting. *Journal of Materials Chemistry A* **9**, 22129-22139 (2021).

9. Lee J, *et al.* Corrosion-engineered bimetallic oxide electrode as anode for high-efficiency anion exchange membrane water electrolyzer. *Chemical Engineering Journal* **420**, (2021).

10. Yu F, *et al.* High-performance bifunctional porous non-noble metal phosphide catalyst for overall water splitting. *Nat Commun* **9**, 2551 (2018).

11. Panda C, Menezes PW, Zheng M, Orthmann S, Driess M. In Situ Formation of Nanostructured Core–Shell Cu3N–CuO to Promote Alkaline Water Electrolysis. *ACS Energy Letters* **4**, 747-754 (2019).

12. Wang X, *et al.* Metal–organic framework derived CoTe2 encapsulated in nitrogen-doped carbon nanotube frameworks: a high-efficiency bifunctional electrocatalyst for overall water splitting. *Journal of Materials Chemistry A* **6**, 3684-3691 (2018).

13. Peng S, *et al.* Necklace-like Multishelled Hollow Spinel Oxides with Oxygen Vacancies for Efficient Water Electrolysis. *J Am Chem Soc* **140**, 13644-13653 (2018).

14. Tang C, Cheng N, Pu Z, Xing W, Sun X. NiSe Nanowire Film Supported on Nickel Foam: An Efficient and Stable 3D Bifunctional Electrode for Full Water Splitting. *Angew Chem Int Ed Engl* **54**, 9351-9355 (2015).

15. Liang K, *et al.* Overall Water Splitting with Room-Temperature Synthesized NiFe Oxyfluoride Nanoporous Films. *ACS Catalysis* **7**, 8406-8412 (2017).

16. Kumar A, Bhattacharyya S. Porous NiFe-Oxide Nanocubes as Bifunctional Electrocatalysts for Efficient Water-Splitting. *ACS Appl Mater Interfaces* **9**, 41906-41915 (2017).

17. Yang Y, *et al.* Self-supported NiFe-LDH@CoSx nanosheet arrays grown on nickel foam as efficient bifunctional electrocatalysts for overall water splitting. *Chemical Engineering Journal* **419**, 129512 (2021).

18. Li H, *et al.* Systematic design of superaerophobic nanotube-array electrode comprised of transition-metal sulfides for overall water splitting. *Nat Commun* **9**, 2452 (2018).

19. Yang Z, *et al.* Trifunctional Self-Supporting Cobalt-Embedded Carbon Nanotube Films for ORR, OER, and HER Triggered by Solid Diffusion from Bulk Metal. *Adv Mater* **31**, e1808043 (2019).

20. Hu B, *et al.* A stable ion-solvating PBI electrolyte enabled by sterically bulky naphthalene for alkaline water electrolysis. *Journal of Membrane Science* **643**, (2022).

21. Lee HI, *et al.* Advanced Zirfon-type porous separator for a high-rate alkaline electrolyser operating in a dynamic mode. *Journal of Membrane Science* **616**, (2020).

22. Diaz LA, Coppola RE, Abuin GC, Escudero-Cid R, Herranz D, Ocón P. Alkali-doped polyvinyl alcohol – Polybenzimidazole membranes for alkaline water electrolysis. *Journal of Membrane Science* **535**, 45-55 (2017).

23. Hu C, *et al.* Robust and durable poly(aryl-co-aryl piperidinium) reinforced membranes for alkaline membrane fuel cells. *Journal of Materials Chemistry A* **10**, 6587-6595 (2022).

24. Kim S, *et al.* Highly selective porous separator with thin skin layer for alkaline water electrolysis. *Journal of Power Sources* **524**, (2022).

25. Aili D, *et al.* Porous poly(perfluorosulfonic acid) membranes for alkaline water electrolysis. *Journal of Membrane Science* **493**, 589-598 (2015).

26. Chen Y, *et al.* Underfocus Laser Induced Ni Nanoparticles Embedded Metallic MoN Microrods as Patterned Electrode for Efficient Overall Water Splitting. *Adv Sci (Weinh)* **9**, e2105869 (2022).

27. Meena A, *et al.* Crystalline-amorphous interface of mesoporous Ni2P @ FePOxHy for oxygen evolution at high current density in alkaline-anion-exchange-membrane water-electrolyzer. *Applied Catalysis B: Environmental* **306**, (2022).

28. López-Fernández E, *et al.* Ionomer-Free Nickel-Iron bimetallic electrodes for efficient anion exchange membrane water electrolysis. *Chemical Engineering Journal* **433**, 133774 (2022).

29. Khataee A, Shirole A, Jannasch P, Krüger A, Cornell A. Anion exchange membrane water electrolysis using Aemion™ membranes and nickel electrodes. *Journal of Materials Chemistry A* **10**, 16061-16070 (2022).

30. Lee J, *et al.* Chemical transformation approach for high-performance ternary NiFeCo metal compound-based water splitting electrodes. *Applied Catalysis B: Environmental* **294**, (2021).

31. Wang X, Lammertink RGH. Dimensionally stable multication-crosslinked poly(arylene piperidinium) membranes for water electrolysis. *Journal of Materials Chemistry A* **10**, 8401-8412 (2022).

32. Chen N, Paek SY, Lee JY, Park JH, Lee SY, Lee YM. High-performance anion exchange membrane water electrolyzers with a current density of 7.68 A cm−2 and a durability of 1000 hours. *Energy & Environmental Science* **14**, 6338-6348 (2021).

33. Li H, Kraglund MR, Reumert AK, Ren X, Aili D, Yang J. Poly(vinyl benzyl methylpyrrolidinium) hydroxide derived anion exchange membranes for water electrolysis. *Journal of Materials Chemistry A* **7**, 17914-17922 (2019).

34. Bhushan M, Mani M, Singh AK, Panda AB, Shahi VK. Self-standing polyaniline membrane containing quaternary ammonium groups loaded with hollow spherical NiCo2O4 electrocatalyst for alkaline water electrolyser. *Journal of Materials Chemistry A* **8**, 17089-17097 (2020).

35. Stiber S, *et al.* A high-performance, durable and low-cost proton exchange membrane electrolyser with stainless steel components. *Energy & Environmental Science* **15**, 109-122 (2022).

36. Kang Z, Chen Y, Wang H, Alia SM, Pivovar BS, Bender G. Discovering and Demonstrating a Novel High-Performing 2D-Patterned Electrode for Proton-Exchange Membrane Water Electrolysis Devices. *ACS Appl Mater Interfaces* **14**, 2335-2342 (2022).

37. Chae JE, *et al.* High-performance multiblock PEMs containing a highly acidic fluorinated-hydrophilic domain for water electrolysis. *Journal of Membrane Science* **638**, (2021).

38. Peng X, *et al.* Insights into Interfacial and Bulk Transport Phenomena Affecting Proton Exchange Membrane Water Electrolyzer Performance at Ultra-Low Iridium Loadings. *Adv Sci (Weinh)* **8**, e2102950 (2021).

39. Choi KJ, Kim H, Kim S-K. Multicomponent nonprecious hydrogen evolution catalysts for high performance and durable proton exchange membrane water electrolyzer. *Journal of Power Sources* **506**, (2021).

40. Stiber S, *et al.* Porous Transport Layers for Proton Exchange Membrane Electrolysis Under Extreme Conditions of Current Density, Temperature, and Pressure. *Advanced Energy Materials* **11**, (2021).

41. Thangavel P, Kim G, Kim KS. Electrochemical integration of amorphous NiFe (oxy)hydroxides on surface-activated carbon fibers for high-efficiency oxygen evolution in alkaline anion exchange membrane water electrolysis. *Journal of Materials Chemistry A* **9**, 14043-14051 (2021).

42. Jang MJ, *et al.* Promotion Effect of Modified Ni/C by La–Ce Oxide for Durable Hydrogen Evolution Reaction. *ACS Sustainable Chemistry & Engineering* **9**, 12508-12513 (2021).

43. Xu Z, Wan L, Liao Y, Wang P, Liu K, Wang B. Anisotropic anion exchange membranes with extremely high water uptake for water electrolysis and fuel cells. *Journal of Materials Chemistry A* **9**, 23485-23496 (2021).

44. Xu D, *et al.* Earth-Abundant Oxygen Electrocatalysts for Alkaline Anion-Exchange-Membrane Water Electrolysis: Effects of Catalyst Conductivity and Comparison with Performance in Three-Electrode Cells. *ACS Catalysis* **9**, 7-15 (2018).

45. Thangavel P, *et al.* Graphene-nanoplatelets-supported NiFe-MOF: high-efficiency and ultra-stable oxygen electrodes for sustained alkaline anion exchange membrane water electrolysis. *Energy & Environmental Science* **13**, 3447-3458 (2020).

46. Yue K, *et al.* In situ ion-exchange preparation and topological transformation of trimetal–organic frameworks for efficient electrocatalytic water oxidation. *Energy & Environmental Science* **14**, 6546-6553 (2021).

47. Lindquist GA, *et al.* Performance and Durability of Pure-Water-Fed Anion Exchange Membrane Electrolyzers Using Baseline Materials and Operation. *ACS Appl Mater Interfaces*, (2021).

48. Cha MS, *et al.* Poly(carbazole)-based anion-conducting materials with high performance and durability for energy conversion devices. *Energy & Environmental Science* **13**, 3633-3645 (2020).

49. Xiao J, *et al.* Water-Fed Hydroxide Exchange Membrane Electrolyzer Enabled by a Fluoride-Incorporated Nickel–Iron Oxyhydroxide Oxygen Evolution Electrode. *ACS Catalysis* **11**, 264-270 (2020).

50. Wang H, Tong Y, Li K, Chen P. Heterostructure engineering of iridium species on nickel/molybdenum nitride for highly-efficient anion exchange membrane water electrolyzer. *J Colloid Interface Sci* **628**, 306-314 (2022).

51. Pavel CC, *et al.* Highly efficient platinum group metal free based membrane-electrode assembly for anion exchange membrane water electrolysis. *Angew Chem Int Ed Engl* **53**, 1378-1381 (2014).

52. Leng Y, Chen G, Mendoza AJ, Tighe TB, Hickner MA, Wang CY. Solid-state water electrolysis with an alkaline membrane. *J Am Chem Soc* **134**, 9054-9057 (2012).

53. Soni R, Miyanishi S, Kuroki H, Yamaguchi T. Pure Water Solid Alkaline Water Electrolyzer Using Fully Aromatic and High-Molecular-Weight Poly(fluorene-alt-tetrafluorophenylene)-trimethyl Ammonium Anion Exchange Membranes and Ionomers. *ACS Applied Energy Materials* **4**, 1053-1058 (2020).

54. Otero J, *et al.* Sulphonated polyether ether ketone diaphragms used in commercial scale alkaline water electrolysis. *Journal of Power Sources* **247**, 967-974 (2014).

55. Wan L, Xu Z, Wang B. Green preparation of highly alkali-resistant PTFE composite membranes for advanced alkaline water electrolysis. *Chemical Engineering Journal* **426**, 131340 (2021).

56. Lee JW, *et al.* Cellulose nanocrystals–blended zirconia/polysulfone composite separator for alkaline electrolyzer at low electrolyte contents. *Chemical Engineering Journal* **428**, 131149 (2022).

57. Schalenbach M, Tjarks G, Carmo M, Lueke W, Mueller M, Stolten D. Acidic or Alkaline? Towards a New Perspective on the Efficiency of Water Electrolysis. *Journal of The Electrochemical Society* **163**, F3197-F3208 (2016).

58. Wan L, Xu Z, Wang P, Liu P-F, Xu Q, Wang B. Dual regulation both intrinsic activity and mass transport for self-supported electrodes using in anion exchange membrane water electrolysis. *Chemical Engineering Journal* **431**, 133942 (2022).

59. Wang L, *et al.* High Performance Anion Exchange Membrane Electrolysis Using Plasma-Sprayed, Non-Precious-Metal Electrodes. *ACS Applied Energy Materials* **2**, 7903-7912 (2019).

60. Faid AY, Barnett AO, Seland F, Sunde S. Tuning Ni-MoO2 Catalyst-Ionomer and Electrolyte Interaction for Water Electrolyzers with Anion Exchange Membranes. *ACS Appl Energy Mater* **4**, 3327-3340 (2021).

61. Li K, *et al.* Engineered Thin Diffusion Layers for Anion-Exchange Membrane Electrolyzer Cells with Outstanding Performance. *ACS Appl Mater Interfaces* **13**, 50957-50964 (2021).

62. Jeon SS, Lim J, Kang PW, Lee JW, Kang G, Lee H. Design Principles of NiFe-Layered Double Hydroxide Anode Catalysts for Anion Exchange Membrane Water Electrolyzers. *ACS Appl Mater Interfaces* **13**, 37179-37186 (2021).

63. Alia SM, Ha M-A, Ngo C, Anderson GC, Ghoshal S, Pylypenko S. Platinum–Nickel Nanowires with Improved Hydrogen Evolution Performance in Anion Exchange Membrane-Based Electrolysis. *ACS Catalysis* **10**, 9953-9966 (2020).

64. Koshikawa H, *et al.* Single Nanometer-Sized NiFe-Layered Double Hydroxides as Anode Catalyst in Anion Exchange Membrane Water Electrolysis Cell with Energy Conversion Efficiency of 74.7% at 1.0 A cm – 2. *ACS Catalysis* **10**, 1886-1893 (2020).

65. Chen N, *et al.* Poly(Alkyl-Terphenyl Piperidinium) Ionomers and Membranes with an Outstanding Alkaline-Membrane Fuel-Cell Performance of 2.58 W cm(-2). *Angew Chem Int Ed Engl* **60**, 7710-7718 (2021).

66. Ham K, Hong S, Kang S, Cho K, Lee J. Extensive Active-Site Formation in Trirutile CoSb2O6 by Oxygen Vacancy for Oxygen Evolution Reaction in Anion Exchange Membrane Water Splitting. *ACS Energy Letters* **6**, 364-370 (2021).

67. Park YS, *et al.* Hierarchical Chestnut-Burr Like Structure of Copper Cobalt Oxide Electrocatalyst Directly Grown on Ni Foam for Anion Exchange Membrane Water Electrolysis. *ACS Sustainable Chemistry & Engineering* **8**, 2344-2349 (2020).

68. Chen P, Hu X. High - Efficiency Anion Exchange Membrane Water Electrolysis Employing Non - Noble Metal Catalysts. *Advanced Energy Materials* **10**, (2020).

69. Kim J-C, Kim J, Park JC, Ahn SH, Kim D-W. Ru2P nanofibers for high-performance anion exchange membrane water electrolyzer. *Chemical Engineering Journal* **420**, (2021).

70. Kang S, Ham K, Lee J. Moderate oxophilic CoFe in carbon nanofiber for the oxygen evolution reaction in anion exchange membrane water electrolysis. *Electrochimica Acta* **353**, (2020).

71. Park JE, *et al.* High-performance anion-exchange membrane water electrolysis. *Electrochimica Acta* **295**, 99-106 (2019).

72. Park YS, *et al.* Co3S4 nanosheets on Ni foam via electrodeposition with sulfurization as highly active electrocatalysts for anion exchange membrane electrolyzer. *International Journal of Hydrogen Energy* **45**, 36-45 (2020).

73. Guo W, Kim J, Kim H, Ahn SH. Cu–Co–P electrodeposited on carbon paper as an efficient electrocatalyst for hydrogen evolution reaction in anion exchange membrane water electrolyzers. *International Journal of Hydrogen Energy* **46**, 19789-19801 (2021).
